# Click estradiol dimers with novel aromatic bridging units: synthesis and anticancer evaluation

**DOI:** 10.1080/14756366.2024.2367139

**Published:** 2024-06-21

**Authors:** Jiří Řehulka, Michal Jurášek, Pavel Dráber, Aleksandra Ivanová, Soňa Gurská, Kateřina Ječmeňová, Olena Mokshyna, Marián Hajdúch, Pavel Polishchuk, Pavel B. Drašar, Petr Džubák

**Affiliations:** a Institute of Molecular and Translational Medicine, Faculty of Medicine and Dentistry, Palacký University, Olomouc, Czech Republic; b Department of Chemistry of Natural Compounds, University of Chemistry and Technology Prague, Praha 6, Czech Republic; c Department of Biology of Cytoskeleton, Institute of Molecular Genetics of the Czech Academy of Sciences, Praha 4, Czech Republic; d Institute of Organic Chemistry and Biochemistry of the Czech Academy of Sciences, Praha 6, Czech Republic; e Laboratory of Experimental Medicine, Institute of Molecular and Translational Medicine, University Hospital Olomouc, Olomouc, Czech Republic

**Keywords:** Estradiol, dimer, tubulin, cancer cell, *in silico*

## Abstract

Estradiol dimers (EDs) possess significant anticancer activity by targeting tubulin dynamics. In this study, we synthesised 12 EDs variants via copper-catalysed azide-alkyne cycloaddition (CuAAC) reaction, focusing on structural modifications within the aromatic bridge connecting two estradiol moieties. *In vitro* testing of these EDs revealed a marked improvement in selectivity towards cancerous cells, particularly for ED1–8. The most active compounds, ED3 (IC_50_ = 0.38 μM in CCRF-CEM) and ED5 (IC_50_ = 0.71 μM in CCRF-CEM) demonstrated cytotoxic effects superior to 2-methoxyestradiol (IC_50_ = 1.61 μM in CCRF-CEM) and exhibited anti-angiogenic properties in an endothelial cell tube-formation model. Cell-based experiments and *in vitro* assays revealed that EDs interfere with mitotic spindle assembly. Additionally, we proposed an *in silico* model illustrating the probable binding modes of ED3 and ED5, suggesting that dimers with a simple linker and a single substituent on the aromatic central ring possess enhanced characteristics compared to more complex dimers.

## Graphical Abstract

**Figure:**
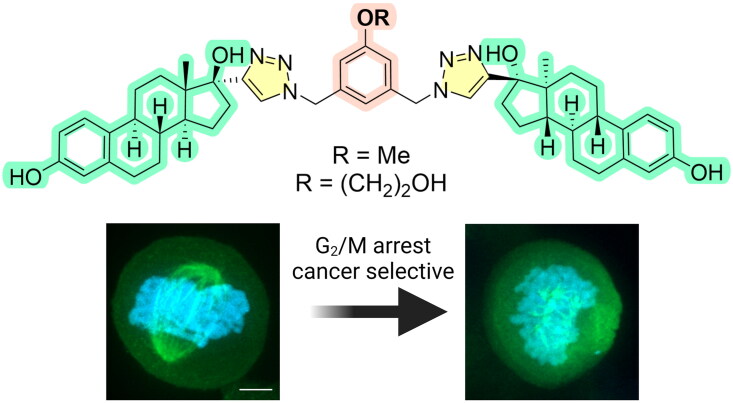

## Introduction

Microtubules are polar dynamic cytoskeletal polymers, formed by αβ-tubulin heterodimers, that can either rapidly grow or disassemble. Microtubule dynamics is essential for many processes within the eukaryotic cells, including the formation of the mitotic spindle, cell motility, and vesicular transport^1^. Microtubules are regulated by microtubule-associated proteins (MAPs), which can affect microtubule dynamics. Additionally, microtubule dynamics may be modulated by microtubule targeting agents (MTAs). The MTAs such as colchicine, nocodazole, and vinca alkaloids prevent tubulin assembly. Conversely, MTAs such as paclitaxel, epothilones, or peloruside A act as microtubule stabilisers^2^. In addition to the effect on the microtubular network, MTAs also modulate mitotic spindle assembly, trafficking on microtubules, and tumour angiogenesis^3^^,^^4^. Targeting microtubule dynamics by small-molecule MTAs has become an important strategy for treating both solid cancers and haematologic malignancies. MTAs are capable of inducing cell cycle arrest and disrupting tumour angiogenesis^5^.

**Figure 1.**
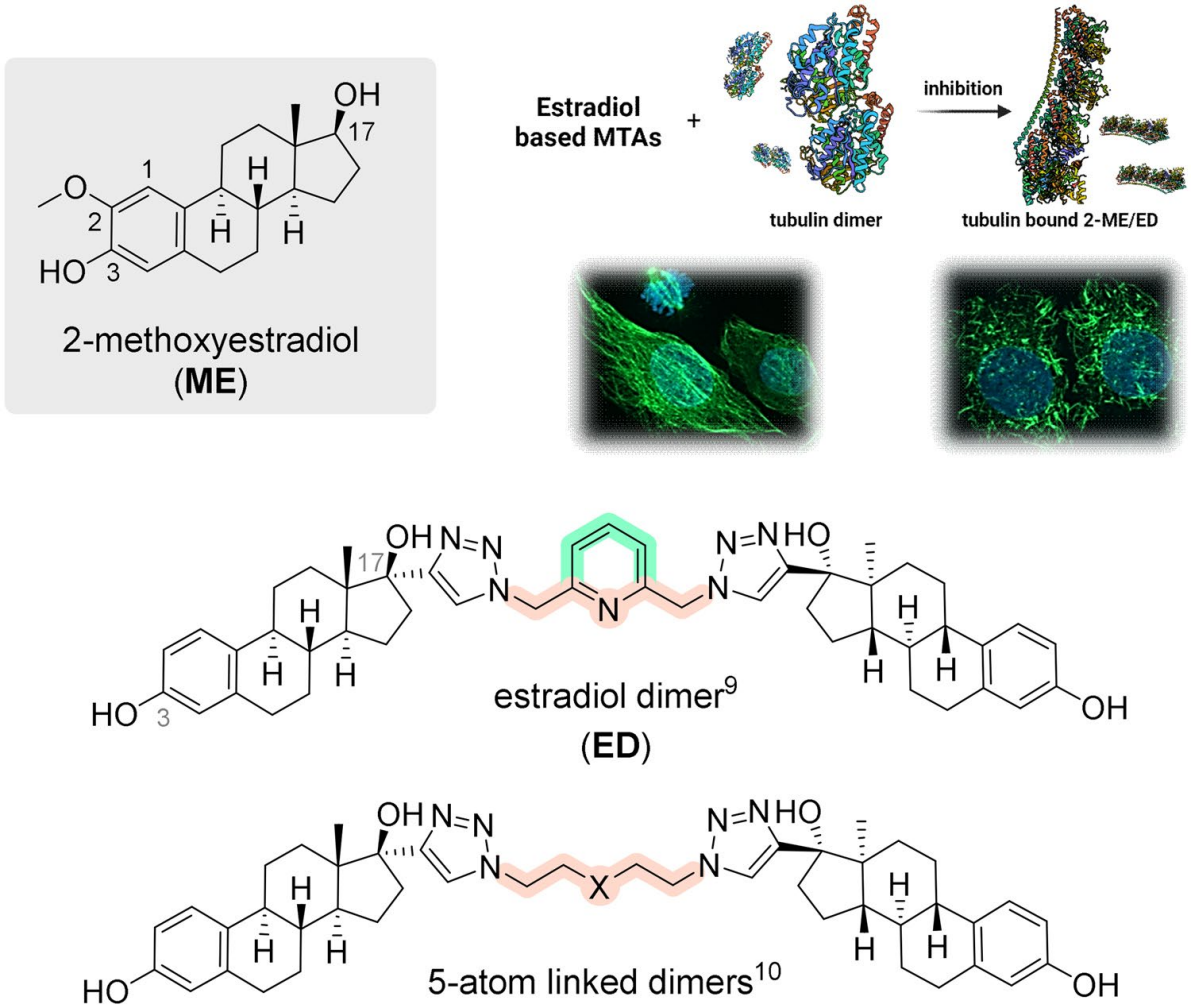
Chemical structure of estradiol-based antimitotics and proposed mechanism of action. Created with Biorender.com.

Interestingly, some steroids and steroid dimers with two steroid moieties within one molecule exhibit significant antiproliferative activities, which are associated with the inhibition of microtubule dynamics (Figure 1). One example is 2-methoxyestradiol (ME), a metabolite of estradiol that is naturally present at low concentrations in human serum. ME has been shown to be an efficient inhibitor of angiogenesis and tumour growth^6^. Several studies have focused on its use in the treatment of various types of haematological malignancies and solid tumours^7^.

Although some steroid dimers are naturally occurring products, the majority of the described dimers are synthetic compounds^8^. In our previous study, we synthesised and evaluated the biological activity of steroidal dimers based on estradiol, 3-*O*-methyl estradiol, testosterone, and pregnenolone, which were bridged by 2,6-bis(azidomethyl)pyridine between D rings^9^. Although the dimerisation of estradiol led to a significant reduction of its oestrogenic and androgenic activity, the study clearly showed strong anticancer activity of estradiol dimer (ED). The effect of ED in cancer cells was associated with the modulation of microtubule dynamics and mitotic arrest. ED significantly prevented the polymerisation of microtubules in U2OS cells. Recently, we compared the biological effect of ED with EDs tethered by diverse five-atom linear linkers or linkers containing substituted aminobenzyl groups^10^. The linker length and substituents proved to be essential for the activity of EDs, and our findings suggested that long linker chains and bulky substituents reduce the activity. Based on this, we speculated that dimers with simple linkers might better fit into the binding site at tubulin than dimers with complex bridges. In this study, we report the synthesis and biological activity of EDs with an aromatic central ring, which is different from the pyridine of the initial ED structure. In addition, we report on ED–protein interactions within the colchicine binding site of tubulin.

## Results and discussion

### Synthesis of dimers

In continuation of our previous work, we prepared another series of steroid dimers by copper-catalysed 1,3-dipolar cycloaddition between the azide groups of the linker and the terminal alkyne group of 17α-ethinyl estradiol (EE)^9^. The synthesised dimers featured the same steroid, i.e. estradiol, however, the original central linker "2,6-bis(triazolylmethylene)pyridine" was replaced with differently substituted aromatics in the same relative arrangement. The reason is to assess the influence of the connecting motif of estradiol units on the overall biological profile of the dimers and thus obtain deeper information about the relationship between the structure and activity of these entities.

**Scheme 1.**
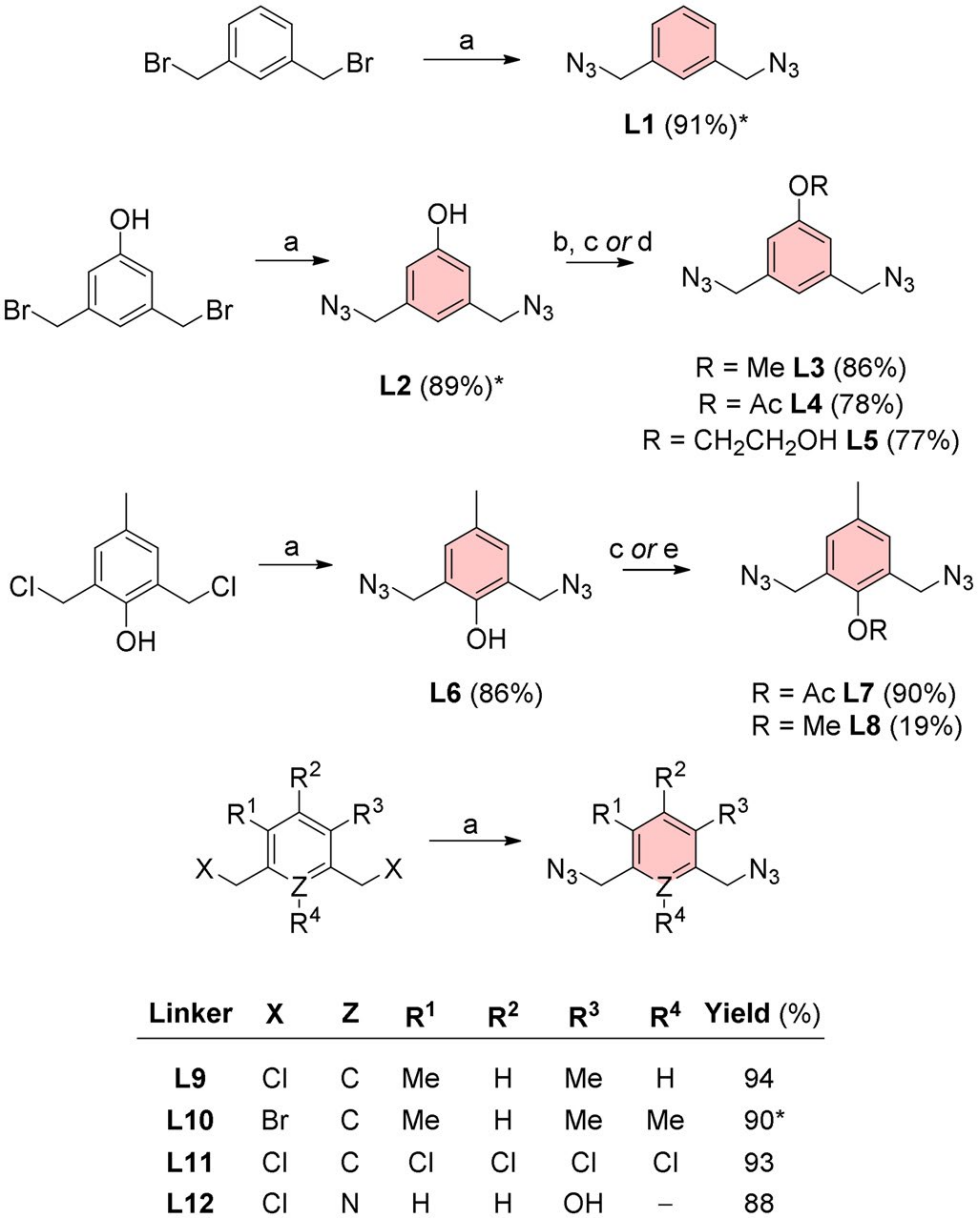
Diazide linkers for CuAAC reaction with EE. Reagents and conditions: (a) NaN_3_, DMF, heated, ON; (b) MeI, NaH, DMF, 0 °C, 4 h; (c) 4-DMAP, Ac_2_O, DCM, RT, ON; (d) acetone, 2-bromoethanol, K_2_CO_3_, 60 °C, ON; (e) MeI, DMF, toluene, NaH, 0 °C, 1 h. *Reaction yields for L1 and L10 reported by Thomas et al.^12^ were 96%. The yield of L2 by Rasheed *et al.*^13^ was 95%.

The azido-terminated bridges (L1–L12) were synthesised via nucleophilic substitution of halogen for the azide group by treatment with sodium azide in *N*,*N*-dimethylformamide (DMF) at elevated temperatures (Scheme 1). The preparation of most diazides proceeded without major difficulties (yields 77–94%) except for the methoxy derivative L8 derived from diazide L6. This reaction proceeded only in very small yields (19%). This poor result is most likely due to phenol ring isomerism under strongly basic conditions. Further experimental details of the synthesis and characterisation of linkers (L1–L12) are provided in Section “Synthesis of linkers”.

**Scheme 2.**
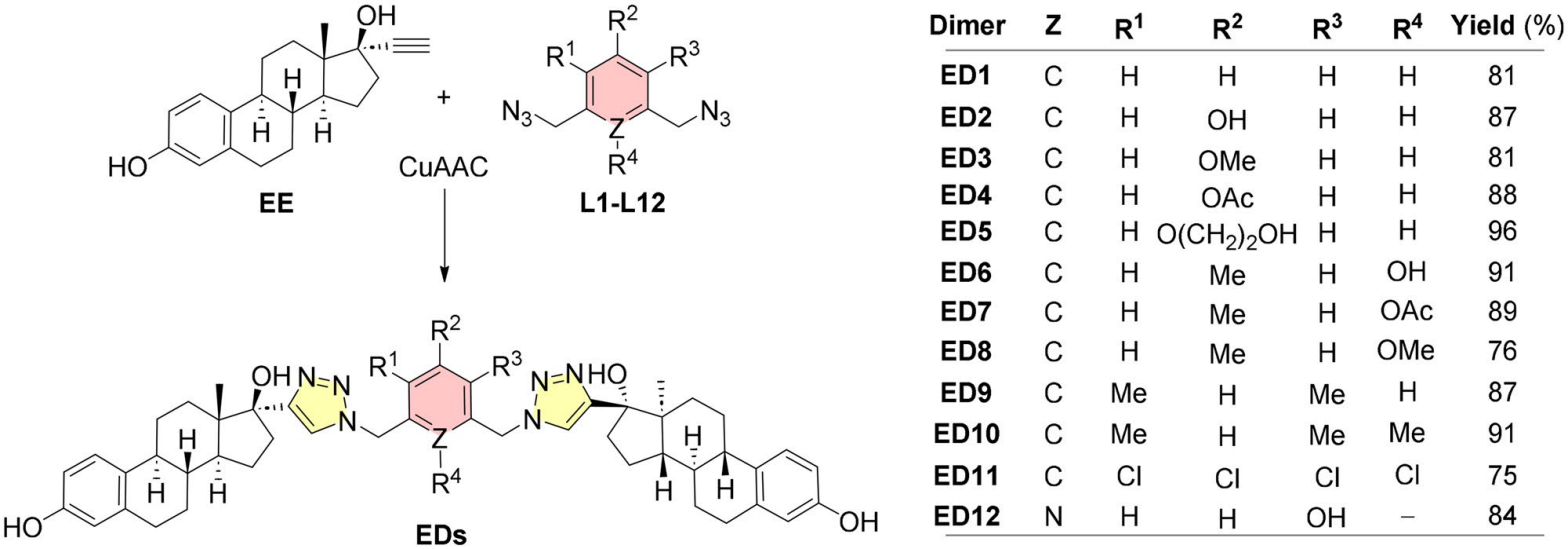
Structures of estradiol dimers prepared by 1,3-dipolar cycloaddition. CuAAC: CuSO_4_·5H_2_O (10 mol%), sodium ascorbate (15 mol%), DMF, MW, 80 °C, 2 h.

Preparation of all new EDs (ED1–12) was performed by microwave (MW)-assisted copper-catalysed azide-alkyne cycloaddition (CuAAC) reaction using typical catalysis operating on the principle of *in situ* reduction of Cu (II) to Cu (I) by sodium ascorbate (Scheme 2). The preparation of new dimers, from prepared diazide aromatics (L1–12; Scheme 1) and EE, proceeded smoothly, and the products were isolated in high yields (75–96%; Scheme 2). A typical signal in the ^1^H NMR spectrum, which confirmed the presence of the expected products, was a singlet of triazole hydrogens between 7.5 and 7.9 ppm and methylene groups of the connecting bridges between 5.5 and 5.9 ppm. Experimental details describing the synthesis of dimers (ED1–12) can be found in Section “Synthesis of estradiol dimers”. Images of ^1^H, ^13^C NMR, HRMS-ESI, and HPLC chromatograms of dimers are thoroughly documented within Supplementary Material (Fig. S1–S48).

### Cytotoxicity

The cytotoxic activity of newly synthesised heterocyclic EDs was evaluated using the MTS assay on a panel of human cancer cell lines (CCRF-CEM, K562, A549, HCT116, HCT116p53−/−, U2OS), their resistant variants (CEM-DNR, K562-TAX), and two normal human cell lines MRC-5 and BJ. The IC_50_ values for estradiol dimers ED1–ED9 were comparable to ME and previously reported ED (Table 1). Cancer-derived cell lines such as CCRF-CEM, K562, and HCT116 showed heightened sensitivity towards these compounds, while the non-malignant human cell lines MRC-5 and BJ did not demonstrate sensitivity to the new dimers, except for ED9 and ED12. This suggests a cancer-specific activity of these compounds. Compounds ED10 and ED11 exhibited no cytotoxic activity across the tested cell lines, while ED12 showed moderate activity. Regarding the epithelial cancer cell lines U2OS and A549, ED4 and ED5 did not exhibit activity, whereas ED2, ED7–ED9, and ED12 were active against at least one of these cell lines. Except for ED10–12, modifications of the central ring, which serves as a coupling unit linking ethynylated steroids, did not significantly affect cytotoxicity. Interestingly, compound ED3, featuring a methoxy moiety in the central ring, displayed submicromolar IC_50_ values across the entire cancer cell panel. In contrast to previously reported dimer ED with pyridine as a coupling unit, newly synthesised ED12 with hydroxy substitution in the pyridine ring displayed reduced cytotoxic activity. In the context of drug resistance, our cell line panel included K562-TAX (subline of K562 resistant to paclitaxel), and CEM-DNR (subline of CCRF-CEM resistant to daunorubicin). The K562-TAX cell line, which overexpresses P-glycoprotein, was sensitive to ED1 and ED8, while other compounds displayed IC_50_ values exceeding the maximum tested concentration of 50 µM. IC_50_ value of ED1 was almost identical in both K562 parental and resistant cell lines in contrast to ED8 which showed a decrease of about one order of magnitude in the resistant subline K562-TAX. Importantly, ED8 maintained its activity against the CEM-DNR cell line, whereas other new dimers did not overcome the acquired chemoresistance, suggesting these dimers may be substrates for drug transporters. To assess the effect of p53 status on compound sensitivity, we employed colorectal carcinoma cell line HCT116 and its p53 deficient counterpart HCT116p53−/−. Both cell lines displayed highly comparable sensitivity to all active compounds. It indicates that EDs could be effective against human cancers with clinically relevant loss of p53. Human umbilical vein endothelial cells (HUVEC) cell line displayed high sensitivity towards the tested set of dimers. The calculated IC_50_ values in HUVEC were used for endothelial cell tube formation experiments.

**Table 1.** Summary of cytotoxic activities (IC_50_, µM).^a^

Compound	CCRF-CEM	CEM-DNR	K562	K562-TAX	A549	HCT116	HCT116p53-	U2OS	MRC-5	BJ	HUVEC
EE	22.5 ± 1.63	22.97 ± 4.7	8.72 ± 2.17	15.6 ± 6.25	31.47 ± 4.16	30.01 ± 6.89	33.57 ± 11.94	22.4 ± 5.65	>50	>50	n/a
ME	1.61 ± 0.097	1.61 ± 0.2	1.43 ± 0.39	1.32 ± 0.2	2.26 ± 0.15	1.58 ± 0.25	1.99 ± 0.41	2.13 ± 0.3	>50	>50	1.0685 ± 0.18
ED	0.49 ± 0.22	>50	0.58 ± 0.23	>50	>50	0.95 ± 0.11	0.9 ± 0.32	6.23 ± 0.7	1.16 ± 0.25	>50	0.0244 ± 0.017
ED1	1.6 ± 0.27	>50	1.63 ± 0.12	1.58 ± 0.16	1.68 ± 0.2	1.41 ± 0.3	1.5 ± 0.32	1.95 ± 0.44	>50	>50	0.0135 ± 0.002
ED2	1.7 ± 0.19	>50	1.48 ± 0.19	>50	6.69 ± 0.79	3.57 ± 0.66	4.26 ± 0.94	>50	>50	>50	<0.0122
ED3	0.38 ± 0.019	>50	0.33 ± 0.074	>50	0.60 ± 0.047	0.47 ± 0.022	0.45 ± 0.039	0.61 ± 0.033	>50	>50	<0.0122
ED4	1.09 ± 0.11	>50	0.49 ± 0.04	>50	>50	2.06 ± 0.18	2.15 ± 0.1	>50	>50	>50	0.0160 ± 0.006
ED5	0.71 ± 0.04	>50	0.42 ± 0.044	>50	>50	2.02 ± 0.19	1.98 ± 0.17	>50	>50	>50	<0.0122
ED6	1.54 ± 0.25	>50	1.39 ± 0.21	>50	8.6 ± 2.22	1.74 ± 0.11	1.87 ± 0.13	5.06 ± 1.68	>50	>50	<0.0122
ED7	1.29 ± 0.13	>50	0.77 ± 0.13	>50	>50	1.61 ± 0.087	1.79 ± 0.17	3.86 ± 1.39	>50	>50	0.014 ± 0.002
ED8	1.38 ± 0.18	3.44 ± 0.69	0.69 ± 0.15	13.08 ± 3.69	>50	1.77 ± 0.16	2.01 ± 0.12	6.93 ± 1.16	>50	>50	0.079 ± 0.048
ED9	1.69 ± 0.057	>50	1.27 ± 0.12	>50	>50	2.02 ± 0.3	1.96 ± 0.26	5.26 ± 1.69	3.2 ± 0.89	>50	0.038 ± 0.011
ED12	6.14 ± 0.93	>50	4.52 ± 0.64	>50	>50	8.78 ± 0.88	10.52 ± 2.48	16.82 ± 3.46	14.2 ± 3.52	11.3 ± 1.41	0.657 ± 0.132
Colchicine	0.011 ± 0.00061	1.02 ± 0.21	0.014 ± 0.002	1.83 ± 0.19	0.047 ± 0.028	0.024 ± 0.0025	0.027 ± 0.0079	0.028 ± 0.014	0.055 ± 0.029	>50	<0.0122

^a^Cytotoxic activity was determined by the MTS assay after a 72-h incubation period. The listed IC_50_ values represent the mean of 3 independent experiments with SD values. Tested cell lines include cancer derived lines such as CCRF-CEM (childhood T-cell acute lymphoblastic leukaemia), CEM-DNR (daunorubicin resistant variant of CCRF-CEM), K562 (chronic myelogenous leukaemia), K562-Tax (paclitaxel-resistant variant of K562), A549 (lung adenocarcinoma), HCT116 (colorectal adenocarcinoma), HCT116p53−/− (p53 gene null variant of HCT116), HeLa (cervical adenocarcinoma), U2OS (osteosarcoma), and normal human cell lines: MRC-5, BJ (normal cycling fibroblasts) and HUVEC (human umbilical vein endothelial cell line). Reference compounds are EE (17α-ethinylestradiol), ME (2-methoxyestradiol), and ED (estradiol dimer ^9^).

When comparing the cytotoxic potency and selectivity of already published dimers with an aliphatic 5-atom linker^10^ vs. ED or current dimers containing a central aromatic motif, it is clear that the presence of an aromatic ring in the linker represents a privileged structural element. In this regard, ED3 seems to be the most potent estradiol-based dimer to date known.

### Cell cycle analysis

**Figure 2.**
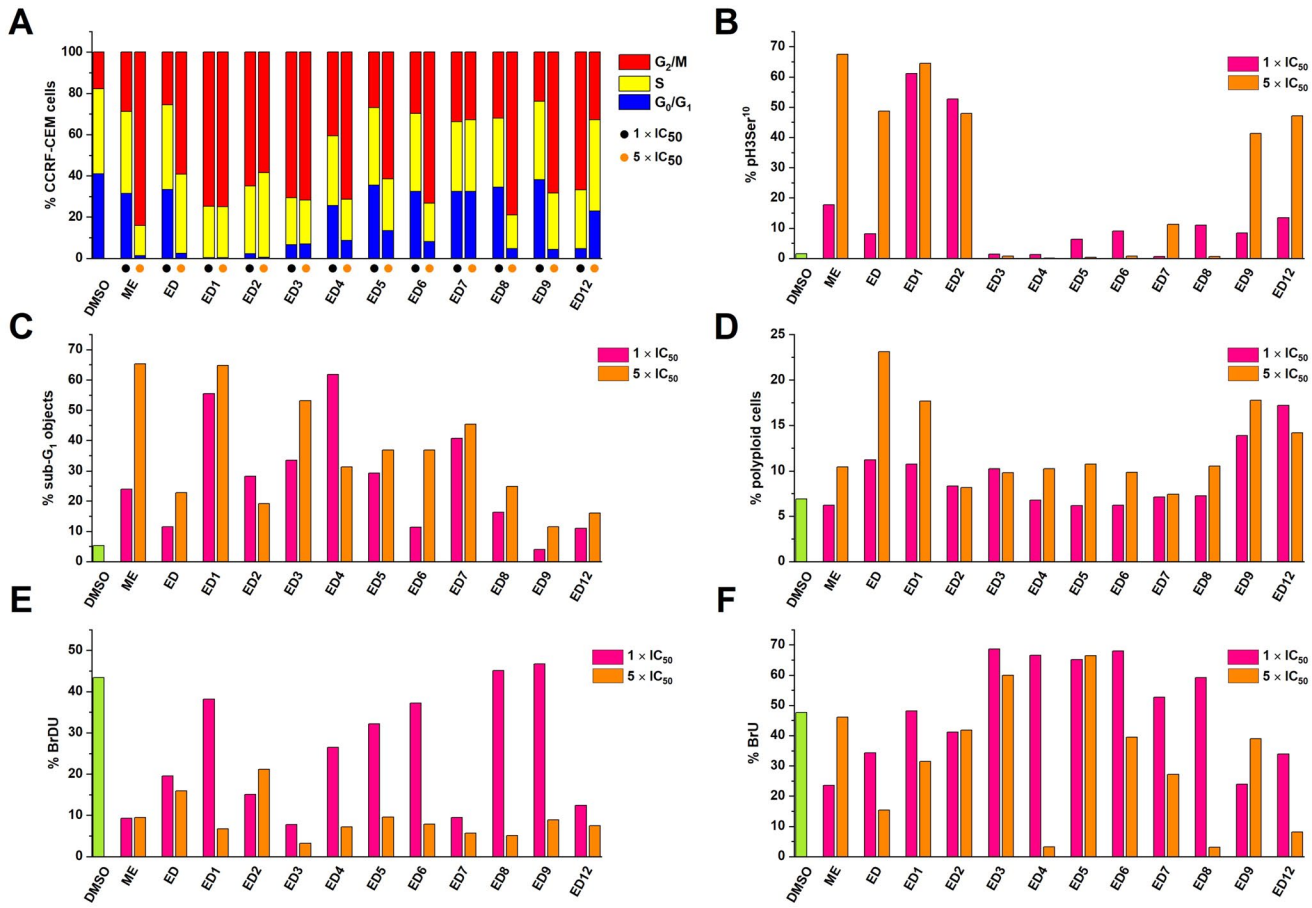
Effect of cytotoxic compounds on cell cycle (A), mitosis (B), sub-G_1_ fraction (C), induction of polyploidy (D), and DNA/RNA synthesis (E, F) as assessed by flow cytometry in CCRF-CEM lymphoblasts (% of positive cells). Experiments were conducted at concentrations corresponding to 1 × IC_50_ and 5 × IC_50_ values. DNA fragmentation was assessed using a logarithmic model expressing the percentage of particles with propidium iodide content lower than cells in the cell cycle’s G_0_/G_1_ phase (<G_1_). Detailed numerical values can be found in the Supplementary Material Table S1.

To further characterise the mode of action of studied compounds, we analysed the cell cycle profile of CCRF-CEM cells treated with compounds for 24 h. The experiment was performed at 1× and 5 × IC_50_ concentrations to observe the effect on the cell cycle. The compounds ED1–ED9 and ED12 induced arrest in G_2_/M phase of the cell cycle (Figure 2(A)). To differentiate between G_2_ block and mitotic arrest, we used phospho-histone H3 (Ser10) mitotic marker (Figure 2(B)). The analysis indicated accumulation of ED1–2 and ED5–12 treated cells in mitosis, while ED3 and ED4 did not induce an increase of mitosis-specific phosphorylation of histone H3. It should be however noted, that ED3 and ED4 showed the highest DNA fragmentation after 24 h incubation and dead cells could affect the mitotic marker analysis. Generally, dimers induced a dose-dependent increase of sub G_1_ cell population with fragmented DNA except for ED2 and ED4 (Figure 2(C)). In addition, the analysis of measured data also revealed a higher number of polyploid cells in a population treated with dimers than in control cell population (Figure 2(D)). To assess the proliferation of treated cells, they were subjected to 5-bromo-2-deoxyuridine (BrDU) labelling. BrDU proliferative marker shows a decrease in the proliferative potential of cells treated with 5 × IC_50_ concentration of all tested compounds (Figure 2(E)). Analogously, changes in 5-bromouridine (BrU) incorporation were examined to monitor overall RNA synthesis. The percentage of BrU-labelled cells was dependent on compound and concentration, nevertheless, ED3, ED5, and ED6 displayed a high rate of RNA synthesis within the gated population of remaining live cells (Figure 2(F)). Taken together the flow cytometry analysis indicates that the main effect of EDs is mitotic block followed by decreased proliferation and eventually cell death.

### In vitro *tubulin assembly*

Supported by cell cycle data and the structural similarity of the newly synthesised EDs with previously reported compounds, we postulated that these novel dimers could potentially modulate microtubule dynamics. To test this hypothesis, we employed a tubulin polymerisation assay, using paclitaxel and colchicine as positive controls for tubulin stabilisers and assembly inhibitors, respectively. Colchicine, a highly active compound that shares its binding site with many MTAs including ME, was used throughout this report and serves as a standard positive control in both *in vitro* and cell-based assays, such as the antiangiogenesis assay presented in the next section.

**Figure 3.**
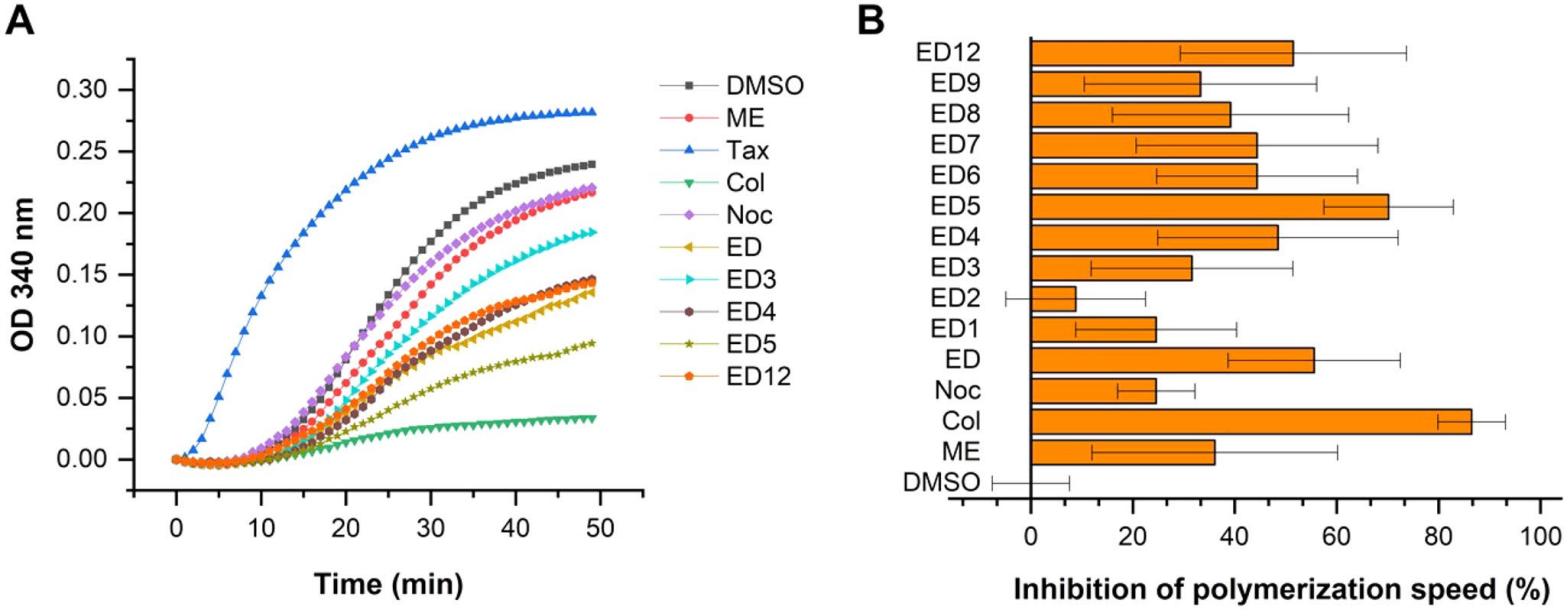
Estradiol dimers inhibit tubulin polymerisation *in vitro* (A). Tubulin assembly was measured using compounds at a concentration of 10 μmol/L, or an equivalent volume of DMSO. Inhibition of polymerisation velocity relative to DMSO control reaction measured at a concentration of 10 μmol/L. Data are represented as mean values with standard deviation (SD) calculated from three independent experiments (B).

Compared to known inhibitors of tubulin polymerisation, the novel compounds ED1, ED3, ED4, ED5, ED6, ED7, ED8, ED9, and ED12 demonstrated comparable or better inhibitory activity against tubulin polymerisation relative to ME, but less potent than colchicine (Figure 3). The most effective structure ED5 ­inhibited the maximal rate of polymerisation (*V*_max_) at slightly lower efficacy than colchicine when both were at equimolar concentrations.

Dimer ED12 with a hydroxyl group at R^3^ was the only tested derivative with the original pyridine ring as the previously published ED. Despite ED12 displayed a reduction in cytotoxic activity as measured by the MTS assay, its inhibitory effect in the tubulin assembly assay was comparable to that of the original ED. A comparison of ED1 and the original ED indicated that the substitution of the central pyridine ring with an aromatic ring resulted in only a marginal reduction in tubulin assembly activity, with cytotoxicity remaining unaltered. Although dimer ED2 with hydroxyl group at R^2^ exerted weak *in vitro* inhibition, other substituents at this position such as methoxy, acetoxy or 2-hydroxyethyl groups (as in ED3–ED5), led to a gradual amplification of the dimers efficacy. Notably, ED5, possessing an ethoxy group at R^2^ was identified as the most potent inhibitor of *in vitro* tubulin polymerisation superior to the original ED. Dimers ED6, ED7, and ED8, all possessing a methyl group at R^2^, displayed comparable activity regardless of the hydroxyl, acetoxy, or methoxy substituent at R^4^. Dimers ED10 and ED11 with multiple methyl or chlorine substituents were inactive. The findings suggest that the central aromatic ring can be further modified without major loss of activity. Specifically, ED3 and ED5, with substituents at R^2^ position, demonstrated the best results in cytotoxicity and *in vitro* assembly assays. Therefore, we performed dose–response tubulin polymerisation assays to compare their activity with colchicine and previously published ED. The polymerisation IC_50_ values of estradiol dimers ED, ED3, and ED5 are highly comparable; however, the dimers are weaker inhibitors than colchicine (Table 2, Fig. S49).

**Table 2.** Inhibition of tubulin assembly in comparison to colchicine as reference compound.

Compound	Colchicine	ED	ED3	ED5
IC_50_ (µmol/L)	1.09 ± 0.33	2.18 ± 1.80	2.24 ± 1.54	2.01 ± 0.21

The listed IC_50_ values with SD represent the mean of two independent experiments.

### Microscopy

**Figure 4.**
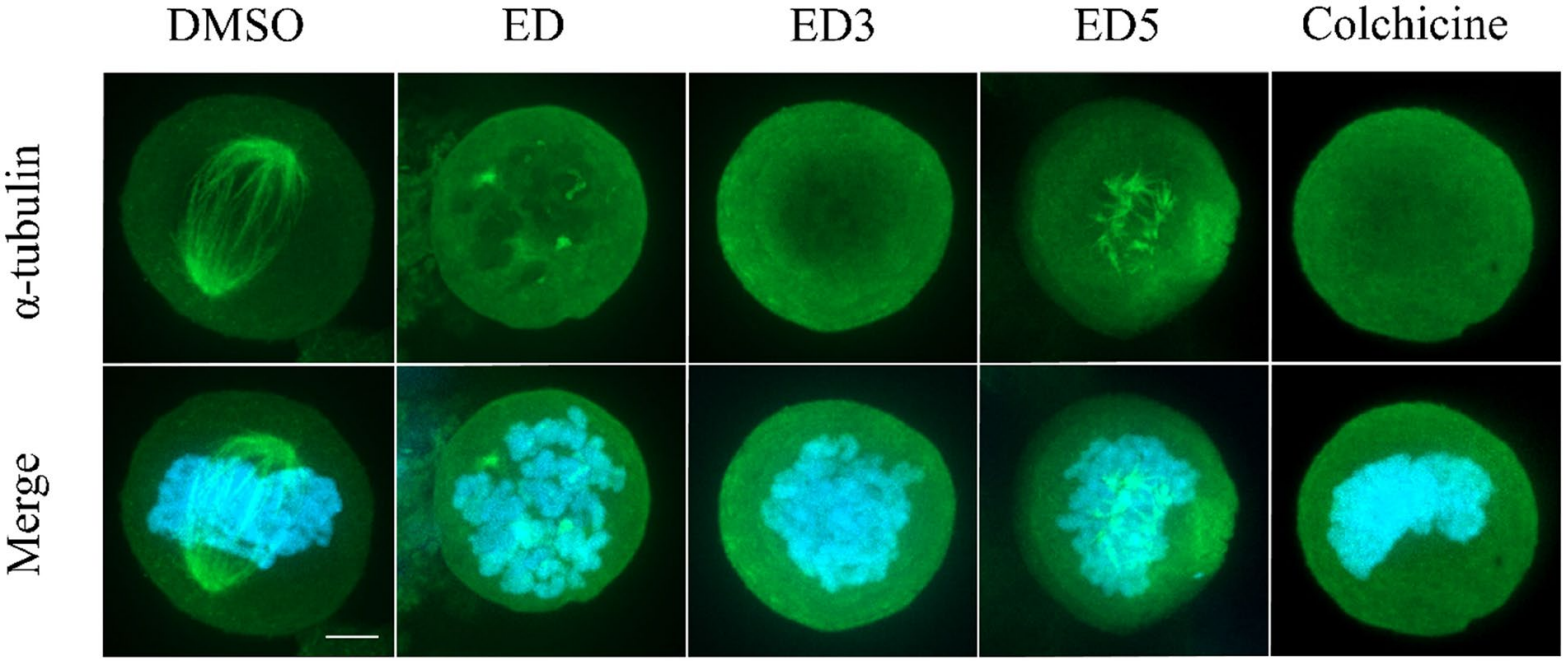
Estradiol dimers inhibit the formation of mitotic spindle. Immunofluorescent imaging of U2OS labelled with α-tubulin – Alexa Fluor-488 antibody (green fluorescence) following a 24-h incubation with DMSO (control), ED (comparative control), ED3, ED5 (all dimers concentrations were set to 10 µM), and colchicine (1 µM). Condensed chromosomes were visualised using Hoechst 33342 (blue fluorescence). Scale bar 2 µm.

To assess the intracellular effects of the most potent dimers, specifically ED3 and ED5, on microtubule assembly, we conducted an examination of microtubule organisation in U2OS cells. Following incubation with either ED, ED3, or ED5, we observed a disruption in microtubule organisation within mitotic cells (Figure 4). Such alterations were manifested as defective mitotic spindles, characterised by short microtubules, or complete absence of the spindle with diffuse tubulin signal as seen in cells treated with the tubulin polymerisation inhibitor, colchicine. In addition, the presence of aster-like tubulin structures indicated the failure in microtubule elongation (Figure 4, ED5). In conclusion, the impaired alignment of spindle microtubules resulted in disorganised chromosomes and aberrant mitotic phenotypes in cells treated with ED, ED3, and ED5. The observed intracellular effects of ED3 and ED5 are consistent with data from *in vitro* assembly assays and cell cycle analyses, thereby strongly supporting the proposed antimitotic mechanisms of these EDs. The phenomenon when ED5 shows activity at the cytoskeletal level in U2OS cells, even when the cytotoxic IC_50_ exceeds 50 μM, can be explained by the inherent differential sensitivity of leukaemia and osteosarcoma cells to tubulin inhibitors. Cytotoxicity IC_50_ values of ED5 for the leukaemia lines CCRF-CEM and K562 are low at 0.71 μM and 0.42 μM, respectively. In contrast, epithelial cell line such as A549 or osteosarcoma U2OS can tolerate polyploidisation for a limited period and thus exhibit elevated IC_50_ values above 50 μM, likely due to the persisting metabolisation of MTS in the viability assay. Importantly, cytotoxic IC_50_ values may vary depending on the duration of exposure to the drug. A more extended exposure could lead to a decrease in the IC_50_ value, and *vice versa*. This temporal factor could also contribute to the observed differential sensitivity among the cell lines. The detailed mechanism and the extent to which these cellular and molecular dynamics (MD) contribute to the observed phenomenon might necessitate further investigation and experimentation. Nevertheless, our findings clearly indicate that both the newly synthesised and previously published EDs and colchicine possess the ability to disrupt the formation of the mitotic spindle, thereby inducing mitotic arrest.

**Figure 5.**
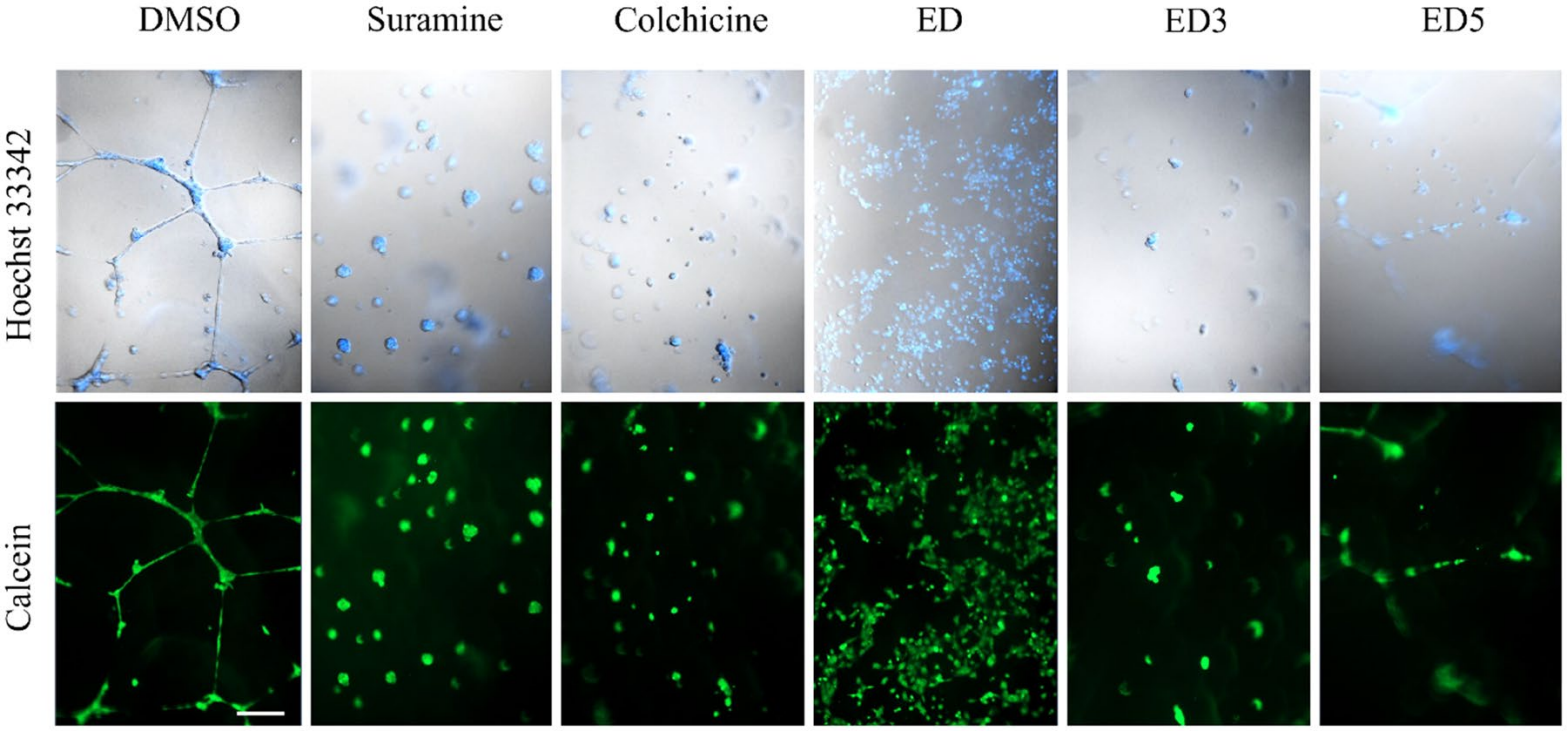
Estradiol dimers inhibit the endothelial cell tube formation angiogenesis. Figure illustrates the network formation of HUVEC primary endothelial cells in the presence of DMSO (control), 60 nM suramine, 60 nM colchicine or 5 × IC_50_ concentration of ED, ED3 and ED5. The cells and nuclei were visualised using DIC and Hoechst 33342 (blue fluorescence) or calcein (green fluorescence). Scale bar: 200 µm.

The effect of MTAs is often associated with their ability to inhibit angiogenesis. To test whether the most potent dimers ED3 and ED5 inhibit the formation of endothelial cell tubes, we utilised the endothelial cell angiogenesis assay. Human umbilical vein endothelial cells cultured on Matrigel formed an endothelial network in control wells (dimethylsulphoxide, DMSO) after 24 h. In contrast, treatment with angiogenesis inhibitors, such as suramine or colchicine, prevented endothelial cell tube formation. A similar effect was observed with ED, ED3, and ED5; however, it was apparent that ED and ED3 had a greater impact on tube formation than ED5 (Figure 5). These findings demonstrate that by inhibiting microtubule formation, our compounds also inhibit angiogenic activity.

### In silico *modelling*

**Figure 6.**
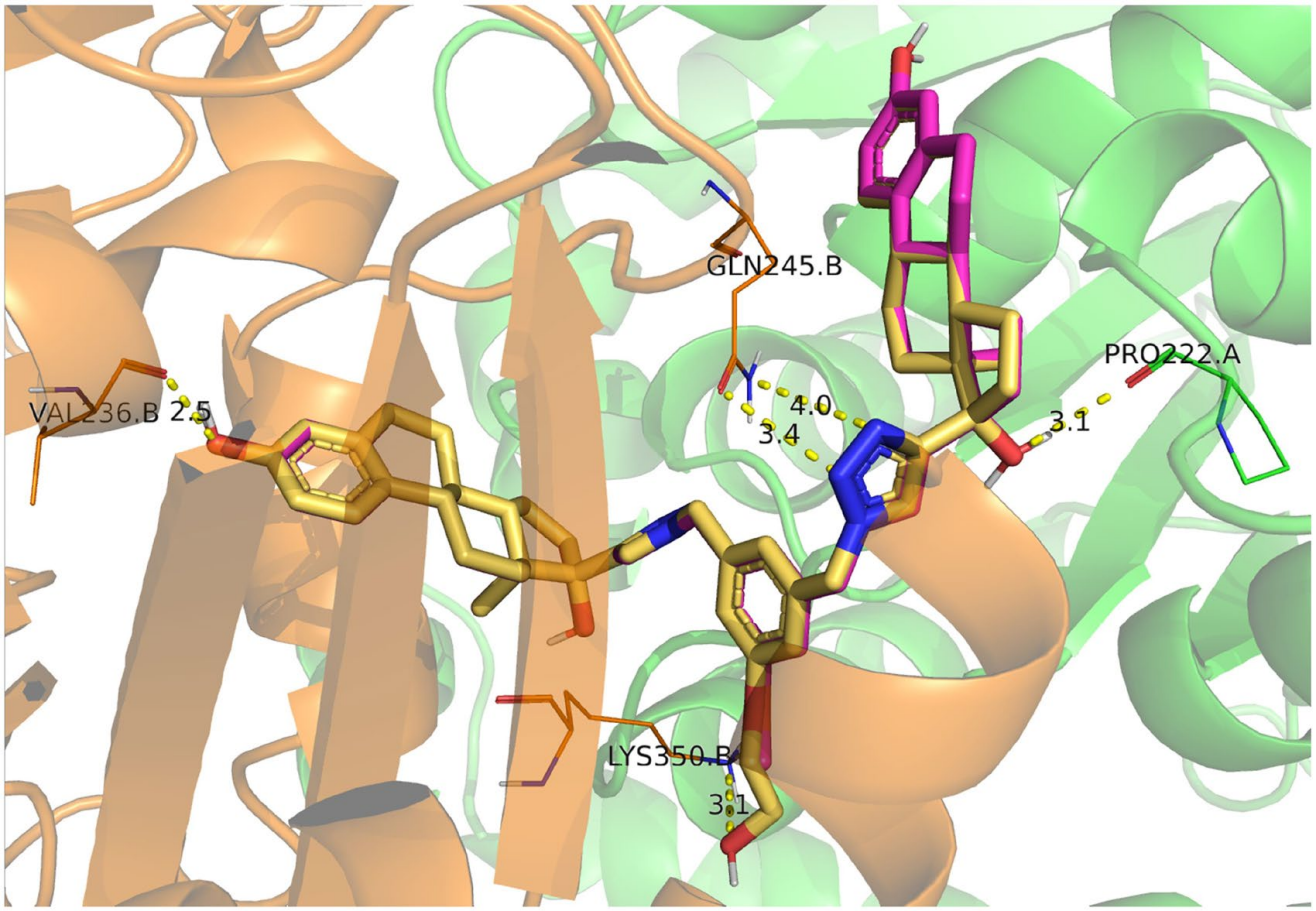
Docking poses of compounds ED3 (magenta) and ED5 (yellow) within the tubulin structure 4O2B (α chain is green, β – orange).

To extend our understanding of ligand tubulin interactions, molecular docking studies were conducted with ED, ED3, and ED5 on two distinct tubulin structures, denoted by their PDB codes as 4O2B and 5LYJ. These structures are complexes of tubulin with colchicine and combretastatin A4, respectively, and were selected due to variations in the conformations of active site residues, which may be important for determining proper ligand binding poses. Upon visual inspection, all compounds failed to dock into the 5LYJ binding site and were docked outside of the cavity. Conversely, for the 4O2B structure, each compound successfully docked into the colchicine binding site. All compounds preserved the same binding mode. One estradiol moiety of the dimers was placed inside the colchicine binding site, specifically in the β-chain of tubulin, and formed H-bond between the hydroxyl group of estradiol and Val236B. The other estradiol moiety binds on the interface between α and β chains. The linker part mainly interacted with β-chain residues. Linkers of ligands comprising polar groups formed H-bonds with side chains of Lys350B or Gln245B (Figure 6).

**Figure 7.**
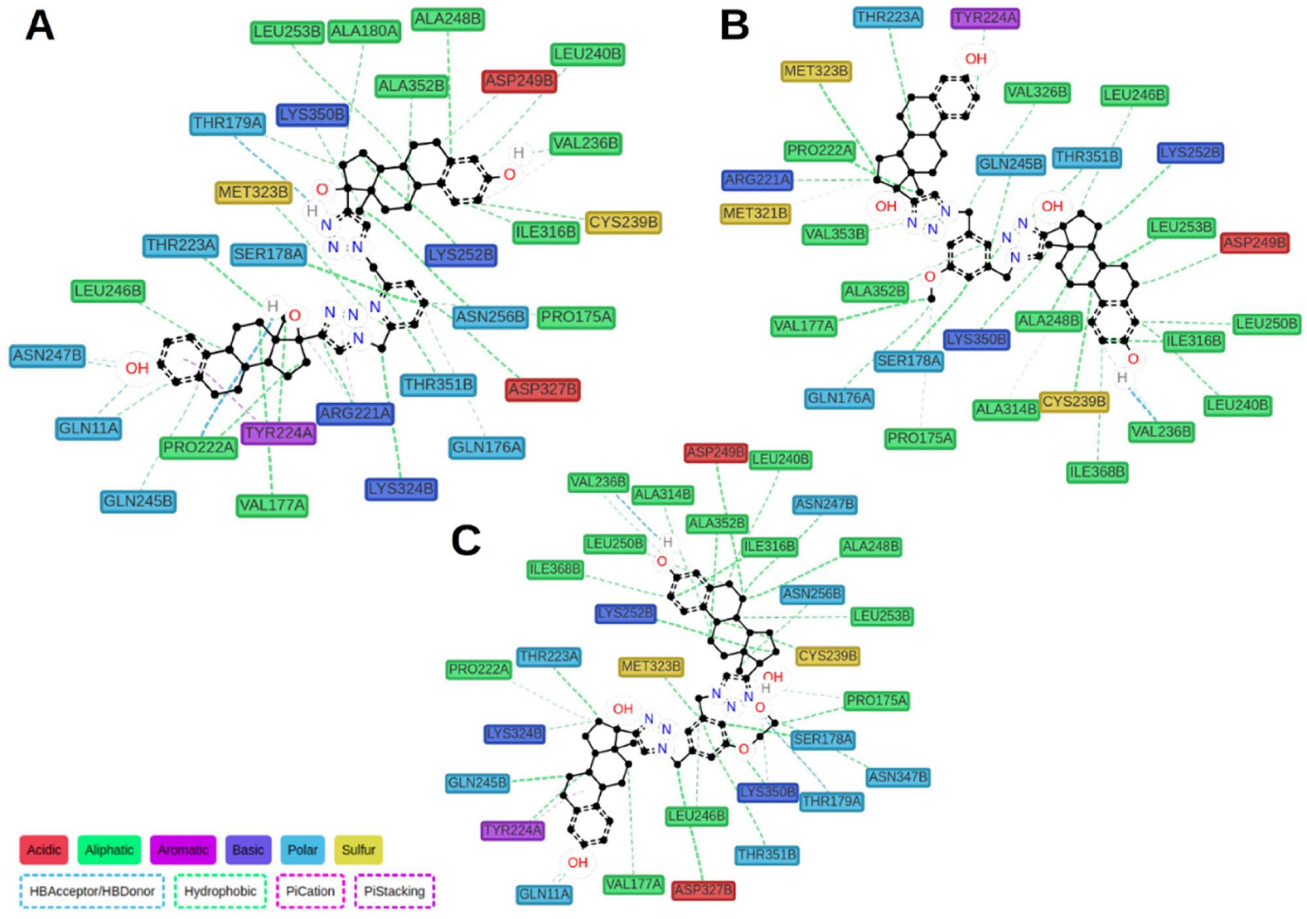
Calculated protein–ligand contacts were identified in at least 10% of MD trajectories for ED (panel A), ED3 (panel B), and ED5 (panel C). Contacts were analysed exclusively for the stable part of trajectories, 50–150 ns.

We performed 150 ns MD simulations for ED5, identified as the most potent tubulin inhibitor, and ED3, as one of the most active compounds in MTS assays, but with moderate tubulin inhibitory activity, and with ED as a reference compound. MD trajectories were stabilised after 50 ns (Fig. S50), thus, we considered for further analysis only the segment within 50–150 ns. The majority of contacts for all ligands identified by ProLIF^11^ were hydrophobic, which is not surprising due to ligand nature (Figure 6). Interaction with Val236B was consistently observed for ED3 and ED5 during the whole simulation, while for the reference compound ED it was observed only for the last 20 ns of the simulation (Fig. S51). In general, a small number of polar contacts were observed for all three compounds, however for ED3, we detected only one H-bond which occurred in at least 10% of time frames. The H-bond between the methoxy group of ED3 and Lys350B, which was observed in docking, was broken from the beginning of MD simulation which suggests its weakness. At the same time, the outer estradiol moiety of ED3 bound between α and β chains started to move outside. The behaviour of ED5 was different. The linker has a longer substituent OCH_2_CH_2_OH, which switched between Lys350B and Thr179A side chains forming an H-bond (Figure 7). The outer estradiol moiety moved deeper between α and β chains and the hydroxyl group of the moiety started to interact with Gln11A residue. ED demonstrated similar behaviour to ED5. Its outer estradiol moiety was also placed deeper and interacted with Gln11A and Asn247B. Since the inner estradiol moiety of ED was shifted relatively to ED5 the ligand at the beginning formed H-bond with Thr147B by the hydroxyl group at position 17 and no contact with Val236B. Later, the ligand moved deeper inside the colchicine site and formed this H-bond simultaneously breaking the contact with Thr147B. This non-deep placement of the outer estradiol moiety of ED3 was the major difference in ligand behaviour and it may explain the lower inhibitory activity of this compound. We repeated MD simulations for ED3 complex two more times with other starting velocities and both times the ligand could not go deeper between α and β chains.

**Figure 8.**
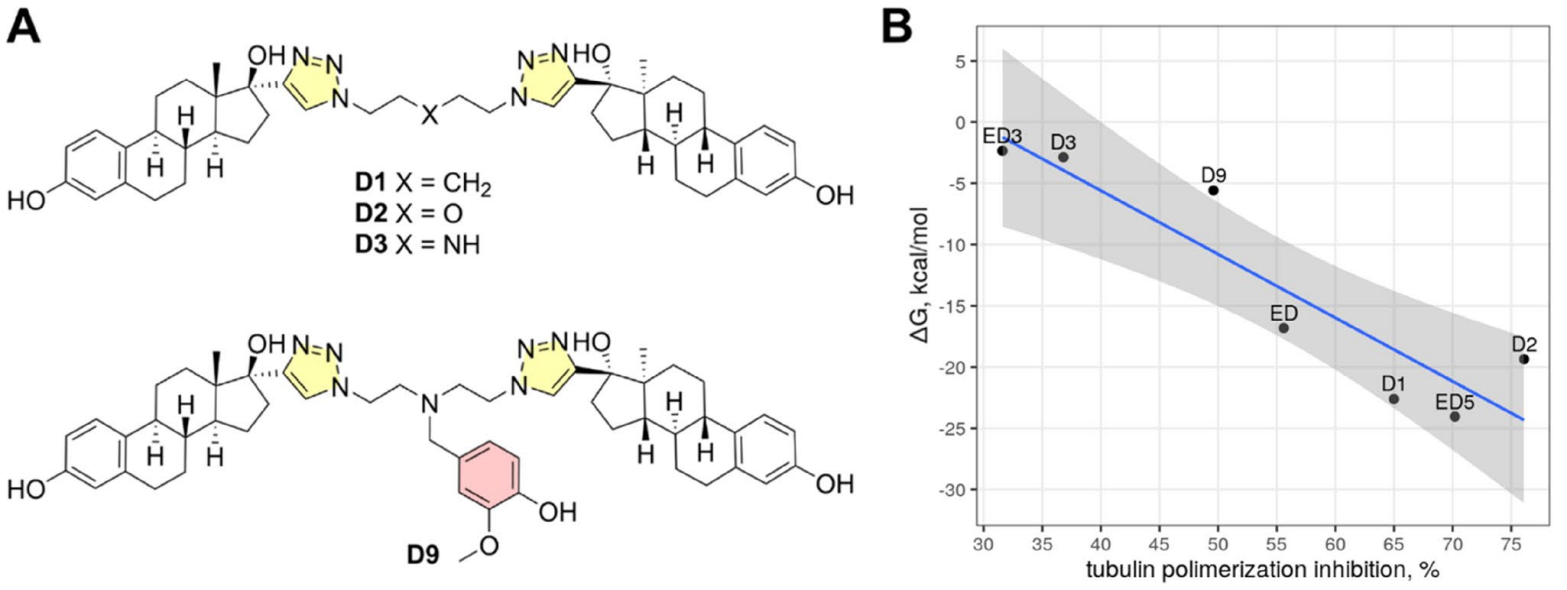
Molecular structures of previously reported estradiol dimers (panel A) and correlation plot between calculated binding free energies and tubulin polymerisation speed (panel B). The shaded region depicts the confidence interval at 0.95 significance level.

To additionally support outputs and conclusions from MD simulations, we computed binding free energy for three selected compounds (ED, ED3, and ED5) using exactly the same MM-PBSA protocol as it was described in our previous publication for computing of binding free energies of estradiol dimmers with aliphatic linkers (Figure 8(A)) to tubulin^10^. To calculate binding free energies, we used the last 10 ns of a whole 150 ns simulation trajectory. The calculated free energies closely corresponded to the measured inhibition of tubulin polymerisation (Figure 8(B), Table 3). Taking into account binding free energies calculated for previously synthesised EDs with aliphatic linkers, the correlation achieved a value 0.92. This supports that identified binding poses can be valid and strengthen our conclusions about protein–ligand interactions.

**Table 3.** Binding free energies of estradiol dimers from this (ED, ED3, ED5) and the previous study (D1, D2, D3, D9)^10^ were calculated by MM-PBSA.

Dimer	Δ*G*, kcal/mol	−*T*Δ*S*, kcal/mol	Tubulin polymerisation inhibition, %
ED	−16.8	16.5	55.6
ED3	−2.4	18.2	31.6
ED5	−24.0	15.0	70.2
D1^a^	−22.6	16.8	65.0
D2^a^	−19.4	13.0	79.1
D3^a^	−2.9	28.8	36.8
D9^a^	−5.6	35.6	49.6

^a^ Values were calculated from Ref. ^10^

## Conclusions

We synthesised a new set of 12 EDs using CuAAC and systematically investigated the impact of the linker on their biological activity, employing a methodology similar to our previous study, to ensure reliable comparability of the results and robust applicability of the SAR analysis. Our findings highlight the crucial role of an appropriate linker, which can either enhance or limit interactions within the colchicine binding site of tubulin. Importantly, our study supports the hypothesis, that simple linkers are generally more favourable than complex ones for this purpose. Dimers with aromatic central ring and multiple substituents showed a loss of activity, whereas dimers with one or two substituents displayed high cytotoxic activity that was comparable with ME. Remarkably, two of the most potent compounds, ED3 and ED5, demonstrated strong cytotoxic effects on various cancer cell lines, at levels comparable to those of ED, a known tubulin inhibitor. Cell-based experiments, as well as *in vitro* assays, proved that these EDs effectively inhibit microtubule dynamics and disrupt mitotic spindle assembly, which aligns well with their observed specific anticancer effects. To strengthen our experimental data, we also performed *in silico* modelling. Our MD simulations supported the docking poses within the pharmacophore and further elucidated the protein–ligand interactions. In our future research, the focus will be placed on the modification of steroid moieties of the dimers to gain deeper SAR information.

## Experimental

### General methods and material

Aluminium silica gel sheets for UV light detection were used for thin layer chromatography (TLC) (TLC silica gel 60 F254, Merck, Kenilworth, NJ). TLCs were visualised by spraying a dilute solution of H_2_SO_4_ in MeOH and the plates were heated on a hot plate. Silica gel 30–60 μm (ICN Biomedicals, Costa Mesa, CA) was used for column chromatography. Agilent-MR DDR2 (Varian, Palo Alto, CA) was used to measure NMR spectra. Chemical shifts are given as *δ* values. For LR-MS and HPLC analyses, Quadrupole LC/MS (ESI ionisation) with an Infinity III LC system (Agilent Technologies, Santa Clara, CA) was used (C18 column: 100 mm; UV detection). The ­following gradient system was used for HPLC: A − 50% MeOH, B − 100% MeOH; 0 min 100% A; 2 min 100% B; 2–16 min 100%; 18 min 50% A; 20 min 100% A. Micro Q-TOF with ESI ionisation (Thermo Scientific, Waltham, MA) was used of HRMS spectra acquisition. Biotage Initiator Classic 355301 (Uppsala, Sweden) was used for MW synthesis. Chemicals were purchased from TCI Europe (Zwijndrecht, Belgium): 4-dimethylaminopyridine − 4-DMAP (>99%), sodium ascorbate (>99%), sodium azide – NaN_3_ (>99%), ethinylestradiol – EE (>98%); from Sigma-Aldrich (St. Louis, MO): cupric sulphate pentahydrate – CuSO_4_·5H_2_O (≥98%), iodomethane – MeI (99.5%), acetic anhydride – Ac_2_O (≥99%), 2,6-bis(chloromethyl)-4-methylphenol (97%), 3-hydroxy-2,6-bis(hydroxymethyl)pyridine hydrochloride (97%), 1,2,3,5-tetrachloro-4,6-bis(chloromethyl)benzene (97%), and abcr GmbH (Karlsruhe, Germany): 1,5-bis(chloromethyl)-2,4-dimethylbenzene (97%). The solvents were supplied by PENTA (Praha, Czech Republic) and used as delivered. Preparation of some diazides was previously described, namely: 1,3-bis(azidomethyl)benzene (L1)^12^, 3,5-bis(azidomethyl)phenol (L2)^13^, and 2,4-bis(azidomethyl)-1,3,5-trimethylbenzene (L10)^12^.

### Synthesis of linkers

#### 1,3-Bis(azidomethyl)-5-methoxybenzene (L3)

To a solution of L2 (250 mg, 1.22 mmol) and MeI (347 mg, 2.45 mmol) in dry DMF (4 mL) at 0 °C NaH (30 mg) was added. The mixture was stirred for 4 h. Then ice-cold water was added and the product was extracted with AcOEt (3 × 30 mL). The combined organic layer was dried over Na_2_SO_4_, filtered and the solvents were evaporated under reduced pressure. The residue was passed through a short plug of silica (hexane–AcOEt, 9:1, *v*/*v*) to obtain L3 (230 mg, 1.05 mmol) as a colourless liquid in 86% yield. *R*_F_ = 0.75 in hexane–AcOEt 5:1 (v/v). ^1^H NMR (400 MHz, CDCl_3_) *δ* ppm: 3.83 (s, 3 H), 4.33 (s, 4 H), 6.84 (s, 2 H), 6.86 (s, 1 H). ^13^C NMR (101 MHz, CDCl_3_) *δ* ppm: 54.49, 55.37, 113.44, 119.88, 137.57, 160.31. LRMS-ESI: for C_9_H_10_N_6_O calcd 218.09 Da, found *m/z* 219.2 [M + H]^+^ and 236.1 [M + NH_4_]^+^.

#### 3,5-Bis(azidomethyl)phenyl acetate (L4)

To a solution of L2 (250 mg, 1.22 mmol) in DCM (15 mL) 4-DMAP (46 mg, 0.37 mmol) and Ac_2_O (0.7 mL) were added. The mixture was stirred overnight after which the mixture was filtered through a plug of silica (hexane–AcOEt, 6:1, v/v) as eluent. The fractions containing the product were collected and solvents were evaporated under reduced pressure. L4 (234 mg, 0.95 mmol) was obtained as a colourless liquid in 78% yield. *R*_F_ = 0.6 in hexane–AcOEt 5:1 (v/v). ^1^H NMR (400 MHz, CDCl_3_) *δ* ppm: 2.28 (s, 3 H), 4.35 (s, 4 H), 7.05 (s, 2 H), 7.12 (s, 1 H). ^13^C NMR (101 MHz, CDCl_3_) *δ* ppm: 20.99, 53.95, 120.91, 124.65, 137.82, 151.21, 169.15. LRMS-ESI: for C_10_H_10_N_6_O_2_ calcd 246.09 Da, found *m*/*z* 264.1 [M + NH_4_]^+^.

#### 2-(3,5-Bis(azidomethyl)phenoxy)ethan-1-ol (L5)

To a solution of L2 (250 mg, 1.22 mmol) in acetone (10 mL) K_2_CO_3_ (337 mg, 2.44 mmol) and 2-bromoethanol (305 mg, 2.44 mmol) were added. The mixture was stirred at 60 °C ON. DCM was added and the mixture was filtered. Solvents were evaporated and the oily residue was chromatographed (hexane–AcOEt, 5:1 → 1:1, v/v) to obtain L5 (233 mg, 0.94 mmol) as a colourless liquid in 77% yield. *R*_F_ = 0.1 in hexane–AcOEt 5:1 (v/v). ^1^H NMR (400 MHz, CDCl_3_) *δ* ppm: 2.65 (s, 1 H), 3.93–3.97 (m, 2 H), 4.06–4.11 (m, 2 H), 4.30 (s, 4 H), 6.84 (s, 2 H), 6.86 (s, 1 H). ^13^C NMR (101 MHz, CDCl_3_) *δ* ppm: 54.40, 61.21, 69.44, 114.00, 120.28, 137.65, 159.36. LRMS-ESI: for C_10_H_12_N_6_O_2_ calcd 248.10 Da, found *m*/*z* ESI^+^ 266.1 [M + NH_4_]^+^, ESI^−^ 293.1 [M + HCOO]^−^.

#### 2,6-Bis(azidomethyl)-4-methylphenol (L6)

To a solution of 2,6-bis(chloromethyl)-4-methylphenol (4 g, 19.5 mmol) in DMF (20 mL), NaN_3_ (4 g, 61.5 mmol) was added. The mixture was stirred for 12 h at 60 °C. The heating was removed and ether (20 mL) was added. The mixture was washed with 0.1 M HCl (50 mL), brine (50 mL), and water (50 mL). The separated organic layer was dried over Na_2_SO_4_, filtered and the solvents were removed under reduced pressure. The residue was chromatographed (hexane–AcOEt, 9:1, v/v) to obtain L6 as a yellow liquid (3.68 g, 16.9 mmol) in 86% yield. *R*_F_ = 0.7 in hexane–AcOEt 5:1 (v/v). ^1^H NMR (400 MHz, CDCl_3_) *δ* ppm: 2.30 (s, 3 H), 4.41 (s, 4 H), 6.30 (br. s., 1 H), 7.01 (s, 2 H). ^13^C NMR (101 MHz, CDCl_3_) *δ* ppm: 20.36, 51.29, 122.36, 130.03, 130.75, 151.14. LRMS-ESI: for C_9_H_10_N_6_O calcd 218.1 Da, found *m/z* 217.1 [M − H]^−^.

#### 2,6-Bis(azidomethyl)-4-methylphenyl acetate (L7)

To a solution of L6 (260 mg, 1.19 mmol) in DCM (5 mL) was added 4-DMAP (146 mg, 1.19 mmol) and Ac_2_O (0.7 mL). The mixture was stirred ON at RT after which the mixture was washed with KHSO_4_ (10%, 2 × 50 mL) and brine (1 × 50 mL). The organic phase was dried over Na_2_SO_4_, filtered and the solvent was evaporated under reduced pressure. The residue was passed through a short plug of silica using hexane–AcOEt as eluent to obtain L7 (281 mg, 1.07 mmol) as a slightly yellowish liquid in 90% yield. R_F_ = 0.7 in hexane–AcOEt 5:1 (v/v). ^1^H NMR (400 MHz, CDCl_3_) *δ* ppm: 2.35 (s, 3 H), 2.36 (s, 3 H), 4.23 (s, 4 H), 7.18 (s, 2 H). ^13^C NMR (101 MHz, CDCl_3_) *δ* ppm: 20.54, 20.83, 50.10, 128.49, 130.75, 136.69, 145.17, 169.08. LRMS-ESI: for C_11_H_12_N_6_O_2_ calcd 260.10 Da, found *m/z* 278.10 [M + NH_4_]^+^.

#### 1,3-Bis(azidomethyl)-2-methoxy-5-methylbenzene (L8)

To a solution of L6 (200 mg, 0.71 mmol) in DMF–toluene (1:1, v/v, 10 mL) at 0 °C, NaH (43 mg) and MeI (302 mg, 2.1 mmol) were added. The mixture was stirred for 1 h after which ether (30 mL) was added and the mixture was washed with KHSO_4_ (10%, 50 mL) and brine (50 mL). The separated organic layer was dried over Na_2_SO_4_, filtered and the solvents were evaporated under reduced pressure. The residue was chromatographed (hexane–AcOEt 10:1, v/v) to obtain L8 (33 mg, 0.14 mmol) in 19% yield. *R*_F_ = 0.8 in hexane–AcOEt, 1:1 (v/v). ^1^H NMR (400 MHz, CDCl_3_) *δ* ppm: 2.37 (s, 3 H), 3.83 (s, 3 H), 4.39 (s, 4 H), 7.15 (s, 2 H). ^13^C NMR (101 MHz, CDCl_3_) *δ* ppm: 20.80, 49.61, 62.72, 128.90, 131.28, 134.47, 154.65. LRMS-ESI: for C_10_H_12_N_6_O calcd 232.11 Da, found *m/z* ESI^+^ 250.1 [M + NH_4_]^+^, ESI^−^ 277.1 [M + HCOO]^−^.

#### 1,5-Bis(azidomethyl)-2,4-dimethylbenzene (L9)

To a solution of 1,5-bis(chloromethyl)-2,4-dimethylbenzene (500 mg, 2.5 mmol) in dry DMF (20 mL), NaN_3_ (650 mg, 10 mmol) was added and the mixture was heated to 80 °C ON. The heating was removed and ether (20 mL) was added. The solids were filtered off. The solvents were evaporated under reduced pressure and the residue was passed through a short plug of silica gel (hexane–AcOEt, 5:1, v/v). L9 (510 mg, 2.35 mmol) was obtained as a slightly yellowish liquid in 94% yield. *R*_F_ = 0.65 in hexane–AcOEt 5:1 (v/v). ^1^H NMR (400 MHz, CDCl_3_) *δ* ppm: 2.36 (s, 6 H), 4.35 (s, 4 H), 7.12 (s, 1 H), 7.18 (s, 1 H). ^13^C NMR (101 MHz, CDCl_3_) *δ* ppm: 18.59, 52.59, 130.46, 131.23, 133.17, 137.17. LRMS-ESI: for C_10_H_12_N_6_ calcd 216.1 Da, found *m*/*z* 217.1 [M + H]^+^ and 234.1 [M + NH_4_]^+^.

#### 1,3-Bis(azidomethyl)-2,4,5,6-tetrachlorobenzene (L11)

To a solution of 1,2,3,5-tetrachloro-4,6-bis(chloromethyl)benzene (500 mg, 1.6 mmol) in dry DMF (20 mL), NaN_3_ (650 mg, 10 mmol) was added and the mixture was heated to 80 °C ON. The heating was removed and ether (20 mL) was added. The solids were filtered off. The solvents were evaporated under reduced pressure and the residue was passed through a short plug of silica gel (hexane–AcOEt 5:1, v/v). L11 (486 mg, 1.49 mmol) was obtained as a slightly yellowish liquid in 93% yield. *R*_F_ = 0.7 in hexane–AcOEt 3:1 (v/v). ^1^H NMR (400 MHz, CDCl_3_) *δ* ppm: 4.76 (s, 4 H). ^13^C NMR (101 MHz, CDCl_3_) *δ* ppm: 50.75, 132.50, 132.64, 135.74, 136.30. LRMS-ESI: for C_8_H_4_Cl_4_N_6_ calcd 323.9 Da, found *m*/*z* 324.9 [M + H]^+^.

#### 2,6-Bis(azidomethyl)pyridine-3-ol (L12)

2,6-Bis(hydroxymethyl)pyridin-3-ol hydrochloride was converted to 2,6-bis(chloromethyl)pyridin-3-ol by previously described method^14^. 2,6-Bis(chloromethyl)pyridin-3-ol (350 mg, 2.26 mmol) was dissolved in dry DMF (10 mL) and NaN_3_ (650 mg, 10 mmol) was added. The mixture was heated to 80 °C ON after which the heating was removed and ether (20 mL) was added. The solids were filtered off and solvents were evaporated under reduced pressure and the residue was passed through a short plug of silica gel (DCM–MeOH, 15:1, v/v). L12 (412 mg, 2.0 mmol) was obtained as a slightly yellowish liquid in 88% yield. *R*_F_ = 0.5 in DCM–MeOH 20:1 (v/v). ^1^H NMR (400 MHz, CD_3_OD) *δ* ppm: 4.35 (s, 2 H), 4.43 (s, 2 H), 7.25 (s, 2 H). ^13^C NMR (101 MHz, CD_3_OD) *δ* ppm: 50.09, 54.26, 123.18, 123.49, 143.14, 145.75, 151.83. LRMS**-**ESI: for C_7_H_7_N_7_O calcd 205.1 Da, found *m*/*z* 206.1 [M + H]^+^ and 223.1 [M + NH_4_]^+^.

### Synthesis of estradiol dimers

*General procedure*: A MW vial (20 mL) was charged with diazide (L1–L12; 1 equiv.) and EE (2.2 equiv.). The mixture was dissolved in dry DMF and aqueous solutions (250 μL) of CuSO_4_·5H_2_O (0.2 equiv.) and sodium ascorbate (0.3 equiv.) were added. The vial was placed onto a MW reactor and stirred 2 h at 80 °C. Solvents were removed under reduced pressure and the residue was purified by column chromatography on silica gel (SiO_2_). The product thus obtained was sonicated with DCM or with a mixture of DCM–Et_2_O. The solids were filtered, washed, and dried *in vacuo.*

#### (17β,17′β)-17,17′-[Benzene-1,3-diylbis(methanediyl-1H-1,2,3-triazole-1,4-diyl)]bisestra-1,3,5(10)-triene-3,17-diol (ED1)

The reaction was performed with L1 (50 mg, 0.27 mmol), EE (173 mg, 0.58 mmol), CuSO_4_·5H_2_O (14 mg, 0.058 mmol), and sodium ascorbate (17 mg, 0.087 mmol) in DMF (3 mL). Chromatography: DCM–MeOH 50:1 → 10:1 (v/v). ED1 (171 mg, 0.22 mmol) was obtained as a white solid in 81% yield. *R*_F_ = 0.21 in DCM–MeOH 20:1 (v/v). ^1^H NMR (400 MHz, DMSO-*d*_6_) *δ* ppm: 0.60 (td, *J* = 12.6, 3.3 Hz, 2 H), 0.91 (s, 6 H), 1.13–1.54 (m, 10 H), 1.58–1.72 (m, 2 H), 1.74–1.86 (m, 6 H), 1.87–1.98 (m, 2 H), 2.04–2.18 (m, 2 H), 2.28–2.41 (m, 2 H), 2.62–2.78 (m, 4 H), 5.12 (s, 2 H), 5.51–5.61 (m, 4 H), 6.41 (d, *J* = 2.3 Hz, 2 H), 6.46 (dd, *J* = 8.6, 2.3 Hz, 2 H), 6.96 (d, *J* = 8.2 Hz, 2 H), 7.24 (d, *J* = 7.8 Hz, 2 H), 7.30 (s, 2 H), 7.39 (t, *J* = 7.6 Hz, 1 H), 7.89 (s, 1 H), 8.97 (s, 1 H); Fig. S1. ^13^C NMR (101 MHz, DMSO-*d*_6_) *δ* ppm: 14.83, 24.03, 26.52, 27.65, 29.73, 33.12, 37.64, 43.62, 47.15, 47.99, 52.84, 81.53, 113.07, 115.30, 123.31, 126.48, 127.86, 127.99, 129.67, 130.87, 137.29, 137.59, 154.95, 155.30; Fig. S2. HRMS-ESI: for C_48_H_56_N_6_O_4_ calcd 780.43630 Da, found *m/z* 781.44385 [M + H]^+^ 803.42682 [M + Na]^+^ and 819.39935 [M + K]^+^; Fig. S3. HPLC: *R*_T_ = 7.379 min; Fig. S4.

#### (17β,17′β)-17,17′-[(5-Hydroxybenzene-1,3-diyl)bis(methanediyl-1H-1,2,3-triazole-1,4-diyl)]bisestra-1,3,5(10)-triene-3,17-diol (ED2)

The reaction was performed with L2 (50 mg, 0.24 mmol), EE (159 mg, 0.54 mmol), CuSO_4_·5H_2_O (13 mg, 0.054 mmol), and sodium ascorbate (16 mg, 0.081 mmol) in DMF (3 mL). Chromatography: CHCl_3_–MeOH 50:1 → 10:1 (v/v). ED2 (164 mg, 0.21 mmol) was obtained as a white solid in 87% yield. *R*_F_ = 0.2 in DCM–MeOH 15:1 (v/v). ^1^H NMR (400 MHz, DMSO-*d*_6_) *δ* ppm: 0.60 (td, *J* = 12.8, 3.7 Hz, 2 H), 0.91 (s, 6 H), 1.20–1.52 (m, 9 H), 1.61–1.71 (m, 2 H), 1.75–1.87 (m, 6 H), 1.87–1.97 (m, 2 H), 2.08–2.16 (m, 2 H), 2.28–2.40 (m, 2 H), 2.63–2.78 (m, 4 H), 5.11 (s, 2 H), 5.47 (s, 4 H), 6.41 (d, *J* = 2.4 Hz, 2 H), 6.46 (dd, *J* = 8.6, 2.4 Hz, 2 H), 6.60 (s, 2 H), 6.72 (s, 1 H), 6.96 (d, *J* = 8.6 Hz, 2 H), 7.85 (s, 2 H), 8.96 (s, 2 H), 9.71 (s, 1 H); Fig. S5. ^13^C NMR (101 MHz, DMSO-*d*_6_) *δ* ppm: 14.83, 24.01, 26.52, 27.65, 29.73, 33.12, 37.62, 43.60, 47.17, 48.00, 52.83, 81.55, 113.08, 114.75, 115.31, 118.24, 123.29, 126.47, 130.89, 137.59, 138.55, 154.88, 155.30, 158.34; Fig. S6. HRMS-ESI: for C_48_H_56_N_6_O_5_ calcd 796.43122 Da, found *m/z* 819.42072 [M + Na]^+^ and 835.39392 [M + K]^+^; Fig. S7. HPLC: *R*_T_ = 7.733 min; Fig. S8.

#### (17β,17′β)-17,17′-[(5-Methoxybenzene-1,3-diyl)bis(methanediyl-1H-1,2,3-triazole-1,4-diyl)]bisestra-1,3,5(10)-triene-3,17-diol (ED3)

The reaction was performed with L3 (115 mg, 0.53 mmol), EE (344 mg, 1.16 mmol), CuSO_4_·5H_2_O (27 mg, 0.11 mmol), and sodium ascorbate (34 mg, 0.17 mmol) in DMF (8 mL). Chromatography: DCM–MeOH 50:1 → 10:1 (v/v). ED3 (350 mg, 0.43 mmol) was obtained as a white solid in 81% yield. *R*_F_ = 0.3 in DCM–MeOH 20:1 (v/v). ^1^H NMR (400 MHz, DMSO-*d*_6_) *δ* ppm: 0.60 (td, *J* = 12.7, 3.5 Hz, 2 H), 0.91 (s, 6 H), 1.14–1.53 (m, 10 H), 1.58–1.70 (m, 2 H), 1.72–1.97 (m, 8 H), 2.04–2.15 (m, 2 H), 2.29–2.41 (m, 2 H), 2.62–2.79 (m, 4 H), 3.68 (s, 3 H), 5.12 (s, 2 H), 5.53 (s, 4 H), 6.41 (d, *J* = 2.4 Hz, 2 H), 6.46 (dd, *J* = 8.4, 2.5 Hz, 2 H), 6.78 (s, 2 H), 6.85 (s, 1 H), 6.96 (d, *J* = 8.6 Hz, 2 H), 7.90 (s, 2 H), 8.96 (s, 2 H); Fig. S9. ^13^C NMR (101 MHz, DMSO-*d*_6_) *δ* ppm: 14.82, 24.03, 26.53, 27.64, 29.73, 33.16, 37.62, 43.65, 47.17, 48.01, 52.78, 55.59, 81.51, 109.99, 113.09, 113.35, 115.31, 119.79, 123.40, 126.46, 130.85, 137.59, 138.79, 154.93, 155.30, 160.12; Fig. S10. HRMS-ESI: for C_49_H_58_N_6_O_5_ calcd 810.44687 Da, found *m/z* 833.43567 [M + Na]^+^ and 849.40891 [M + K]^+^; Fig. S11. HPLC: *R*_T_ = 7.425 min; Fig. S12.

#### 3,5-Bis({4-[(17β)-3,17-dihydroxyestra-1,3,5(10)-trien-17-yl]-1H-1,2,3-triazol-1-yl}methyl)phenyl acetate (ED4)

The reaction was performed with L4 (138 mg, 0.56 mmol), EE (365 mg, 1.23 mmol), CuSO_4_·5H_2_O (30 mg, 0.12 mmol), and sodium ascorbate (36 mg, 0.18 mmol) in DMF (8 mL). Chromatography: DCM–MeOH 100:1 → 20:1 (v/v). ED4 (412 mg, 0.49 mmol) was obtained as a white solid in 88% yield. *R*_F_ = 0.3 in DCM–MeOH 20:1 (v/v). ^1^H NMR (400 MHz, DMSO-*d*_6_) *δ* ppm: 0.59 (td, *J* = 12.7, 3.5 Hz, 2 H), 0.91 (s, 6 H), 1.14–1.52 (m, 10 H), 1.53–1.68 (m, 2 H), 1.73–1.87 (m, 6 H), 1.88–1.99 (m, 2 H), 2.04–2.13 (m, 2 H), 2.14 (s, 3 H), 2.30–2.42 (m, 2 H), 2.62–2.79 (m, 4 H), 5.13 (s, 2 H), 5.60 (s, 4 H), 6.41 (d, *J* = 2.4 Hz, 2 H), 6.46 (dd, *J* = 8.4, 2.5 Hz, 2 H), 6.92 (s, 2 H), 6.96 (d, *J* = 8.2 Hz, 2 H), 7.21 (s, 1 H), 7.92 (s, 2 H), 8.95 (s, 2 H); Fig. S13. ^13^C NMR (101 MHz, DMSO-*d*_6_) *δ* ppm: 14.80, 21.05, 24.01, 26.52, 27.64, 29.72, 33.15, 37.56, 43.62, 47.15, 48.02, 52.32, 81.49, 113.07, 115.32, 121.04, 123.52, 124.98, 126.44, 130.87, 137.59, 138.98, 151.27, 154.92, 155.30, 169.35; Fig. S14. HRMS-ESI: for C_50_H_58_N_6_O_6_ calcd 838.44178 Da, found *m/z* 839.44939 [M + H]^+^ 861.43132 [M + Na]^+^ and 877.40411 [M + K]^+^; Fig. S15. HPLC: *R*_T_ = 7.429 min; Fig. S16.

#### (17β,17′β)-17,17′-{[5-(2-Hydroxyethoxy)benzene-1,3-diyl]bis(methanediyl-1H-1,2,3-triazole-1,4-diyl)}bisestra-1,3,5(10)-triene-3,17-diol (ED5)

The reaction was performed with L5 (70 mg, 0.28 mmol), EE (183 mg, 0.62 mmol), CuSO_4_·5H_2_O (15 mg, 0.062 mmol), and sodium ascorbate (18 mg, 0.093 mmol) in DMF (6 mL). Chromatography: CHCl_3_–MeOH 50:1 → 20:1 (v/v). ED5 (230 mg, 0.27 mmol) was obtained as a white solid in 96% yield. *R*_F_ = 0.4 in DCM–MeOH 10:1 (v/v). ^1^H NMR (400 MHz, DMSO-*d*_6_) *δ* ppm: 0.60 (td, *J* = 12.8, 3.3 Hz, 2 H), 0.91 (s, 6 H), 1.14–1.51 (m, 11 H), 1.60–1.70 (m, 2 H), 1.72–1.99 (m, 9 H), 2.05–2.17 (m, 2 H), 2.29–2.40 (m, 2 H), 2.61–2.78 (m, 4 H), 3.64 (q, *J* = 5.4 Hz, 2 H), 3.84–3.94 (m, 2 H), 4.86 (t, *J* = 5.5 Hz, 1 H), 5.12 (s, 2 H), 5.53 (s, 4 H), 6.41 (d, *J* = 2.4 Hz, 2 H), 6.46 (dd, *J* = 8.4, 2.5 Hz, 2 H), 6.77 (s, 2 H), 6.86 (s, 1 H), 6.97 (d, *J* = 8.6 Hz, 2 H), 7.90 (s, 2 H), 8.95 (s, 2 H); Fig. S17. ^13^C NMR (101 MHz, DMSO-*d*_6_) *δ* ppm: 14.83, 24.04, 26.52, 27.65, 29.73, 33.17, 37.64, 43.64, 47.17, 48.01, 52.79, 59.84, 70.02, 81.53, 113.09, 113.80, 115.31, 119.72, 123.38, 126.47, 130.88, 137.59, 138.75, 154.95, 155.30, 159.56; Fig. S18. HRMS-ESI: for C_50_H_60_N_6_O_6_ calcd 840.45743 Da, found *m/z* 841.46474 [M + H]^+^ 863.44643 [M + Na]^+^ and 879.41920 [M + K]^+^; Fig. S19. HPLC: *R*_T_ = 7.382 min; Fig. S20.

#### (17β,17′β)-17,17′-[(2-Hydroxy-5-methylbenzene-1,3-diyl)bis(methanediyl-1H-1,2,3-triazole-1,4-diyl)]bisestra-1,3,5(10)-triene-3,17-diol (ED6)

The reaction was performed with L6 (75 mg, 0.34 mmol), EE (224 mg, 0.76 mmol), CuSO_4_·5H_2_O (19 mg, 0.076 mmol), and sodium ascorbate (23 mg, 0.11 mmol) in DMF (8 mL). Chromatography: CHCl_3_–MeOH 100:1 → 20:1 (v/v). ED6 (258 mg, 0.31 mmol) was obtained as a white solid in 91% yield. *R*_F_ = 0.25 in DCM–MeOH 20:1 (v/v). ^1^H NMR (400 MHz, DMSO-*d*_6_) *δ* ppm: 0.61 (td, *J* = 12.7, 3.5 Hz, 2 H), 0.91 (s, 6 H), 1.12–1.55 (m, 12 H), 1.58–1.70 (m, 2 H), 1.72–1.98 (m, 9 H), 2.08 (s, 3 H), 2.30–2.40 (m, 2 H), 2.62–2.79 (m, 5 H), 5.11 (s, 2 H), 5.56 (s, 2 H), 6.41 (d, *J* = 2.4 Hz, 2 H), 6.46 (dd, *J* = 8.4, 2.5 Hz, 2 H), 6.71 (s, 2 H), 6.95 (d, *J* = 8.6 Hz, 2 H), 7.80 (s, 2 H), 8.96 (s, 2 H), 9.30 (s, 1 H); Fig. S21. ^13^C NMR (101 MHz, DMSO-*d*_6_) *δ* ppm: 14.82, 20.44, 24.02, 26.56, 27.65, 29.72, 33.12, 37.55, 43.70, 47.18, 48.01, 48.64, 81.54, 113.11, 115.33, 123.34, 124.85, 126.40, 129.28, 130.16, 130.82, 137.60, 150.37, 154.73, 155.33; Fig. S22. HRMS-ESI: for C_49_H_58_N_6_O_5_ calcd 810.44687 Da, found *m/z* 811.45428 [M + H]^+^, 833.43593 [M + Na]^+^ and 849.40846 [M + K]^+^; Fig. S23. HPLC: *R*_T_ = 7.731 min; Fig. S24.

#### 2,6-Bis({4-[(17β)-3,17-dihydroxyestra-1,3,5(10)-trien-17-yl]-1H-1,2,3-triazol-1-yl} methyl)-4-methylphenyl acetate (ED7)

The reaction was performed with L7 (100 mg, 0.38 mmol), EE (224 mg, 0.85 mmol), CuSO_4_·5H_2_O (21 mg, 0.085 mmol), and sodium ascorbate (24 mg, 0.12 mmol) in DMF (10 mL). Chromatography: AcOEt. ED7 (280 mg, 0.33 mmol) was obtained as a white solid in 89% yield. *R*_F_ = 0.25 in DCM–MeOH 20:1 (v/v). ^1^H NMR (400 MHz, DMSO-*d*_6_) *δ* ppm 0.63 (td, *J* = 12.8, 3.7 Hz, 2 H), 0.92 (s, 6 H), 1.14–1.52 (m, 10 H), 1.59–1.70 (m, 2 H), 1.73–1.99 (m, 8 H), 2.04–2.14 (m, 2 H), 2.22 (s, 3 H), 2.34 (s, 3 H), 2.62–2.79 (m, 4 H), 5.00 (s, 2 H), 5.44 (s, 4 H), 6.42 (d, *J* = 2.4 Hz, 2 H), 6.47 (dd, *J* = 8.4, 2.5 Hz, 2 H), 6.96 (d, *J* = 8.6 Hz, 2 H), 6.99 (s, 2 H), 7.78 (s, 2 H), 8.87 (s, 2 H); Fig. S25. ^13^C NMR (101 MHz, DMSO-*d*_6_) *δ* ppm: 14.79, 20.78, 21.00, 23.98, 26.59, 27.66, 29.68, 33.07, 37.57, 43.68, 47.23, 48.05, 48.47, 81.55, 113.14, 115.38, 123.37, 126.33, 129.55, 130.54, 130.91, 136.31, 137.61, 144.77, 154.86, 155.35, 169.12; Fig. S26. HRMS-ESI: for C_51_H_60_N_6_O_6_ calcd 852.45743 Da, found *m/z* 853.46466 [M + H]^+^, 875.44659 [M + Na]^+^ and 891.41945 [M + K]^+^; Fig. S27. HPLC: *R*_T_ = 7.642 min; Fig. S28.

#### (17β,17′β)-17,17′-[(2-Methoxy-5-methylbenzene-1,3-diyl)bis(methanediyl-1H-1,2,3-triazole-1,4-diyl)]bisestra-1,3,5(10)-triene-3,17-diol (ED8)

The reaction was performed with L8 (80 mg, 0.34 mmol), EE (221 mg, 0.75 mmol), CuSO_4_·5H_2_O (19 mg, 0.075 mmol), and sodium ascorbate (22 mg, 0.11 mmol) in DMF (10 mL). Chromatography: CHCl_3_–AcOEt 1:1. ED8 (211 mg, 0.26 mmol) was obtained as a white solid in 76% yield. *R*_F_ = 0.4 in DCM–AcOEt 1:1 (v/v). ^1^H NMR (400 MHz, DMSO-*d*_6_) *δ* ppm: 0.60 (td, *J* = 12.7, 3.5 Hz, 2 H), 0.91 (s, 6 H), 1.14–1.53 (m, 10 H), 1.58–1.69 (m, 2 H), 1.70–1.87 (m, 6 H), 1.88–1.98 (m, 2 H), 2.03–2.11 (m, 2 H), 2.13 (s, 3 H), 2.30–2.41 (m, 2 H), 2.62–2.78 (m, 4 H), 3.73 (s, 3 H), 5.12 (s, 2 H), 5.59 (s, 4 H), 6.41 (d, *J* = 2.4 Hz, 2 H), 6.47 (dd, *J* = 8.6, 2.4 Hz, 2 H), 6.80 (s, 2 H), 6.95 (d, *J* = 8.6 Hz, 2 H), 7.88 (s, 2 H), 8.96 (s, 2 H); Fig. S29. ^13^C NMR (101 MHz, DMSO-*d*_6_) *δ* ppm: 14.99, 20.93, 24.20, 26.71, 27.84, 29.88, 33.34, 37.72, 43.91, 47.38, 48.14, 48.22, 62.53, 81.70, 113.30, 115.52, 123.82, 126.57, 130.22, 130.46, 130.98, 134.24, 137.79, 153.88, 155.00, 155.52; Fig. S30. HRMS-ESI: for C_50_H_60_N_6_O_5_ calcd 824.46252 Da, found *m/z* 847.45156 [M + Na]^+^ and 863.42470 [M + K]^+^; Fig. S31. HPLC: *R*_T_ = 7.703 min; Fig. S32.

#### 17,17′-[(4,6-Dimethyl-1,3-phenylene)bis(methylene-1H-1,2,3-triazole-1,4-diyl)]di[estra-1,3,5(10)-triene-3,17β-diol] (ED9)

The reaction was performed with L9 (50 mg, 0.23 mmol), EE (151 mg, 0.51 mmol), CuSO_4_·5H_2_O (13 mg, 0.051 mmol), and sodium ascorbate (15 mg, 0.077 mmol) in DMF (6 mL). Chromatography: CHCl_3_–AcOEt 1:1. ED9 (162 mg, 0.20 mmol) was obtained as a white solid in 87% yield. *R*_F_ = 0.5 in DCM–AcOEt 1:1 (v/v). ^1^H NMR (400 MHz, DMSO-*d*_6_) *δ* ppm: 0.58 (br t, *J* = 12.3 Hz, 2 H), 0.90 (br s, 6 H), 1.13–1.50 (m, 10 H), 1.61–1.72 (m, 2 H), 1.72–1.86 (m, 5 H), 1.91 (br t, *J* = 12.5 Hz, 2 H), 2.07 (br d, *J* = 12.1 Hz, 2 H), 2.22 (s, 6 H), 2.28–2.40 (m, 2 H), 2.50 (br s, 1 H), 2.61–2.77 (m, 4 H), 5.08 (s, 2 H), 5.45–5.55 (m, 4 H), 6.40 (br s, 2 H), 6.45 (br d, *J* = 8.2 Hz, 2 H), 6.93 (br d, *J* = 8.6 Hz, 2 H), 7.07 (br d, *J* = 17.6 Hz, 2 H), 7.68 (s, 2 H), 8.96 (s, 2 H); Fig. S33. ^13^C NMR (101 MHz, DMSO-*d*_6_) *δ* ppm: 14.79, 18.59, 24.00, 26.51, 27.62, 29.69, 33.07, 37.60, 43.61, 47.15, 47.97, 51.16, 55.32, 81.51, 113.03, 115.28, 122.87, 126.42, 130.81, 132.37, 133.18, 137.21, 137.54, 154.73, 155.27; Fig. S34. HRMS-ESI: for C_50_H_60_N_6_O_4_ calcd 808.46760 Da, found *m/z* 809.47452 [M + H]^+^, 831.45659 [M + Na]^+^ and 847.42983 [M + K]^+^; Fig. S35. HPLC: *R*_T_ = 7.778 min; Fig. S36.

#### 17,17′-[(2,4,6-Trimethyl-1,3-phenylene)bis(methylene-1H-1,2,3-triazole-1,4-diyl)]di[estra-1,3,5(10)-triene-3,17β-diol] (ED10)

The reaction was performed with L10 (50 mg, 0.22 mmol), EE (142 mg, 0.48 mmol), CuSO_4_·5H_2_O (12 mg, 0.048 mmol), and sodium ascorbate (14 mg, 0.072 mmol) in DMF (5 mL). Chromatography: CHCl_3_–AcOEt 1:1. ED10 (165 mg, 0.20 mmol) was obtained as a white solid in 91% yield. *R*_F_ = 0.6 in DCM–AcOEt 1:1 (v/v). ^1^H NMR (400 MHz, DMSO-*d*_6_) *δ* ppm: 0.52 (br t, *J* = 12.5 Hz, 2 H), 0.88 (br s, 6 H), 1.13–1.48 (m, 14 H), 1.61–1.94 (m, 12 H), 2.02–2.12 (m, 2 H), 2.24–2.32 (m, 2 H), 2.35 (br s, 6 H), 2.38 (br s, 3 H), 2.68 (br s, 4 H), 5.05 (br s, 2 H), 5.53–5.65 (m, 4 H), 6.41 (br s, 2 H), 6.43–6.49 (m, 2 H), 6.95 (br d, *J* = 8.2 Hz, 2 H), 7.05 (br s, 1 H), 7.59 (br s, 2 H), 8.97 (br s, 2 H); Fig. S37. ^13^C NMR (101 MHz, DMSO-*d*_6_) *δ* ppm: 14.81, 16.05, 20.12, 24.00, 26.45, 27.66, 29.69, 32.94, 37.58, 43.60, 47.15, 47.97, 48.15, 81.51, 113.05, 115.29, 122.58, 126.42, 130.82, 130.87, 131.00, 137.57, 138.77, 154.35, 155.30; Fig. S38. HRMS-ESI: for C_51_H_62_N_6_O_4_ calcd 822.48325 Da found *m/z* 823.49054 [M + H]^+^, 845.47238 [M + Na]^+^ and 861.44532 [M + K]^+^; Fig. S39. HPLC: *R*_T_ = 7.889 min; Fig. S40.

#### 17,17′-[(2,4,5,6-Tetrachloro-1,3-phenylene)bis(methylene-1H-1,2,3-triazole-1,4-diyl)]di[estra-1,3,5(10)-triene-3,17β-diol] (ED11)

The reaction was performed with L11 (50 mg, 0.24 mmol), EE (159 mg, 0.54 mmol), CuSO_4_·5H_2_O (13 mg, 0.054 mmol), and sodium ascorbate (16 mg, 0.081 mmol) in DMF (5 mL). Chromatography: CHCl_3_–MeOH 25:1 (v/v). ED11 (161 mg, 0.18 mmol) was obtained as a white solid in 75% yield. *R*_F_ = 0.35 in DCM–MeOH 25:1 (v/v). ^1^H NMR (400 MHz, DMSO-*d*_6_) *δ* ppm: 0.53 (br t, *J* = 12.5 Hz), 0.88 (br s, 6 H), 1.08–1.49 (m, 14 H), 1.55–1.66 (m, 2 H), 1.75 (br d, *J* = 12.9 Hz, 6 H), 1.89 (br t, *J* = 11.9 Hz, 2 H), 2.05 (dt, *J* = 7.5, 1.3 Hz, 2 H), 2.21–2.35 (m, 2 H), 2.60–2.75 (m, 4 H), 5.10 (br s, 2 H), 5.82–5.89 (m, 4 H), 6.38 (br s, 2 H), 6.43 (br d, *J* = 7.8 Hz, 2 H), 6.93 (br d, *J* = 7.8 Hz, 2 H), 7.89 (br s, 2 H), 8.94 (br s, 2 H); Fig. S41. ^13^C NMR (101 MHz, DMSO-*d*_6_) *δ* ppm: 14.80, 23.97, 26.46, 27.65, 29.66, 32.93, 37.61, 43.57, 47.18, 47.96, 50.49, 81.48, 113.04, 115.29, 123.59, 126.41, 130.79, 132.76, 136.50, 137.55, 154.45, 155.28; Fig. S42. HRMS-ESI: for C_48_H_52_Cl_4_N_6_O_4_ calcd 916.28041 Da found *m/z* 919.28503 [M + H]^+^, 941.26660 [M + Na]^+^ and 957.24005 [M + K]^+^; Fig. S43. HPLC: *R*_T_ = 7.781 min; Fig. S44.

#### 17,17′-[(3-Hydroxypyridine-2,6-diyl)bis(methylene-1H-1,2,3-triazole-1,4-diyl)]di[estra-1,3,5(10)-triene-3,17β-diol] (ED12)

The reaction was performed with L12 (40 mg, 0.19 mmol), EE (127 mg, 0.42 mmol), CuSO_4_·5H_2_O (10 mg, 0.042 mmol), and sodium ascorbate (12 mg, 0.063 mmol) in DMF (4 mL). Chromatography: CHCl_3_–MeOH 25:1 → 10:1 (v/v). ED12 (131 mg, 0.16 mmol) was obtained as a white solid in 84% yield. *R*_F_ = 0.4 in DCM–MeOH 10:1 (v/v). ^1^H NMR (400 MHz, DMSO-*d*_6_) *δ* ppm: 0.59 (td, *J* = 13.1, 3.9 Hz, 1 H), 0.68 (td, *J* = 13.1, 3.9 Hz, 1 H), 0.91 (s, 6 H), 1.13–1.53 (m, 10 H), 1.56–1.69 (m, 2 H), 1.71–1.98 (m, 7 H), 2.01–2.15 (m, 2 H), 2.29–2.43 (m, 2 H), 2.60–2.79 (m, 4 H), 5.11 (s, 2 H), 5.50 (s, 2 H), 5.58 (s, 2 H), 6.40 (s, 2 H), 6.45 (td, *J* = 8.0, 2.4 Hz, 2 H), 6.93 (d, *J* = 8.6 Hz, 1 H), 6.96 (d, *J* = 8.6 Hz, 1 H), 7.09 (d, *J* = 8.2 Hz, 1 H), 7.27 (d, *J* = 8.2 Hz, 1 H), 7.77 (s, 1 H), 7.81 (s, 1 H), 8.95 (br. s., 2 H), 10.55 (br. s., 1 H); Fig. S45. ^13^C NMR (101 MHz, DMSO-*d*_6_) *δ* ppm: 14.83, 24.02, 26.52, 27.63, 29.72, 32.99, 37.46, 37.58, 43.61, 47.17, 47.98, 50.47, 54.26, 81.57, 113.07, 115.30, 123.41, 123.67, 123.98, 126.46, 130.88, 137.58, 142.14, 145.51, 151.55, 154.37, 154.73, 155.29; Fig. S46. HRMS-ESI: for C_47_H_55_N_7_O_5_ calcd 797.42647 Da, found *m/z* 798.43362 [M + H]^+^, 820.41585 [M + Na]^+^ and 836.38804 [M + K]^+^; Fig. S47. HPLC: *R*_T_ = 7.502 min; Fig. S48.

### Cell culture

T-lymphoblastic leukaemia cell line CCRF-CEM, chronic myelogenous leukaemia cell line K562, lung carcinoma cell line A549, colorectal carcinoma cell line HCT116, osteosarcoma cell line U2OS and non-tumour skin fibroblasts BJ and MRC-5 cell lines were purchased from the American Type Culture Collection (ATCC). The p53 deficient variant, HCT116p53−/−, was purchased from Horizon Discovery (Cambridge, UK). The daunorubicin-resistant CCRF-CEM cell line CEM-DNR bulk and paclitaxel-resistant K562-TAX sublines were developed in-house by exposing parental cell lines to escalating concentrations of daunorubicin and paclitaxel, respectively. Cells were maintained in the recommended culture medium (Sigma-Aldrich, St. Louis, MO) supplemented with 10% foetal calf serum, 100 U/mL penicillin, and 100 μg/mL streptomycin. The HUVEC line was obtained from PromoCell (Heidelberg, Germany). The cells were cultured in endothelial cell growth medium with SupplementMix for endothelial cells (PromoCell, Heidelberg, Germany). The incubation conditions were a humidified atmosphere of 95% air and 5% CO_2_ at 37 °C^10^.

### Cell viability and cytotoxicity assays

Cells were seeded in 384-well plates and after overnight incubation treated with compounds. All tested compounds were dissolved in 100% DMSO, and fourfold serial dilutions were performed using an Echo550 liquid handler (Labcyte, San Jose, CA). The experiments were performed in technical duplicates and three biological replicates. Cells were incubated with compounds for 72 h and thereafter 5 μL of MTS reagent was added to each well. After a 3 h incubation, absorbance was measured at 540 nm using an Envision plate reader (PerkinElmer, Waltham, MA). IC_50_ values were calculated from dose − response curves using Dotmatics software^10^.

### FACS analysis

Treated CCRF-CEM cells were harvested, washed with ice-cold phosphate-buffered saline (PBS), fixed in cold 70% ethanol and stored at −20 °C. Prior to analysis, fixed cells were permeabilised with 0.25% Triton X-100 in PBS for 15 min, blocked in 1% bovine serum, and incubated with anti-phospho-Histone H3 (Ser^10^) antibody (Merck Millipore, Burlington, MA). Subsequently, the cells were treated with 50 μg/mL RNAse, stained with propidium iodide and analysed by FACSCalibur (Becton Dickinson, Franklin Lakes, NJ) flow cytometer at 488 nm. Cell cycle distribution (G_1_, S, and G_2_/M) was analysed using ModFitLT software (Verity, Topsham, ME), and reflects only viable population prior to the fixing procedure. Particles with lower propidium iodide content than G_1_ phase cells were classified as non-viable cells with fragmented DNA. Flow cytometry data visualisation was performed using OriginPro 2018b (b9.5.5.409) software (OriginLab Corporation, Northampton, MA)^10^.

#### BrDU incorporation analysis

Thirty minutes before harvesting, cells were pulse-labelled with 10 μM BrDU. After overnight fixation in ice-cold 70% ethanol, cells were resuspended in 2 M HCl for 30 min at room temperature (RT), then washed with 0.1 M Na_2_B_4_O_7_ and blocked with PBS containing 0.5% Tween-20 and 1% BSA. BrDU incorporation was analysed using anti-BrdU antibody clone MoBu-1 (Exbio, Vestec, Czech Republic) and a secondary anti-mouse-FITC antibody (Sigma-Aldrich, St. Louis, MO). Cells were washed with PBS and stained with 0.1 mg/mL propidium iodide and 0.5 mg/mL RNase A for 1 h at RT and analysed by flow cytometry using a 488 nm single beam laser (FACSCalibur, Becton Dickinson, Franklin Lakes, NJ). The percentage of cells with incorporated BrDU was analysed using CellQuest software^10^.

#### BrU incorporation analysis

Prior to the trypsinisation, cells were treated with 1 mM BrU for 30 min. Subsequently, the cells were fixed in 1% paraformaldehyde with 0.05% of NP-40 for 15 min at RT and stored overnight at 4 °C. Fixed cells were washed with 1% glycine in PBS and stained with primary anti-BrdU antibody clone MoBu-1 crossreacting with BrU (Exbio, Vestec, Czech Republic) for 30 min at RT, washed with PBS and labelled with secondary anti-mouse-FITC antibody (Sigma-Aldrich, St. Louis, MO). Following 1-h incubation in 1% paraformaldehyde containing 0.05% NP-40, cells were stained with propidium iodide (0.1 mg/mL) and treated with RNase A (0.5 mg/mL) for 1 h at RT. The percentage of BrU positive cells was quantified using CellQuest software^10^.

### Tubulin polymerisation assay

Tubulin polymerisation assay kit (Cytoskeleton, Denver, CO) was used according to the manufacturer’s instructions. Polymerisation of porcine brain tubulin (>99% purity) was measured using an EnVision Multilabel Plate Reader (PerkinElmer, Waltham, MA) at 37 °C, in the presence of either 10 μmol/L test compounds or DMSO. Maximum polymerisation velocity (*V*_max_) was calculated from the polymerisation curves. Data visualisation was performed in OriginPro 2018b (b9.5.5.409) software (OriginLab Corporation, Northampton, MA)^10^.

### Immunofluorescence

U2OS cells, cultivated on coverslips, underwent a PBS wash and were then fixed in a solution of 3% paraformaldehyde in a buffer containing 10 mM MES, 150 mM NaCl, 5 mM EGTA, 5 mM MgCl_2_, 5 mM glucose, adjusted to a pH of 6.1. The nuclei were made visible using Hoechst 33342. Subsequently, a 60-min blocking step with 5% goat serum in PBS (SpinChem, Seattle, WA) was followed by a 60-min incubation with α-tubulin (DM1A) mouse monoclonal antibody (Cell Signaling, Danvers, MA) in PBS supplemented with 1% BSA and 0.3% Triton X-100. Visualisation was achieved using Alexa Fluor-488 conjugated anti-mouse antibodies (Life Technologies, Carlsbad, CA). Between each step, the samples underwent a series of three 5-min washes with PBS. Finally, the samples were mounted using Vectashield Mounting Medium with DAPI and imaged utilising a Zeiss spinning disk confocal microscope equipped with CSU-X1 unit (Yokogawa, Musashino, Japan)^10^.

### Endothelial cell tube formation angiogenesis assay

The experiment was performed in 96-well plates pre-coated with 50 μL of growth factor reduced basement membrane matrix Matrigel (Corning, Corning, NY). The coating was set by incubating the plates for 1 h at 37 °C. Subsequently, HUVEC cells were seeded at a density of 15 000 cells per well, and dissolved test substances were added to this layer in a total volume of 100 μL. The assay plates were then incubated for 24 h to facilitate endothelial tube formation. After this incubation, the cells were stained with Hoechst 33342 (Thermo Fisher Scientific, Waltham, MA) to a final concentration of 10 μM and calcein AM (Invitrogen, Carlsbad, CA) to a final concentration of 2 µg/mL for 30 min, followed by visualisation in DIC and fluorescent modes using a Zeiss fluorescent microscope (Oberkochen, Germany).

### In silico *modelling*

For computational analyses, 3D complexes (4O2B, 5LYJ) of bovine tubulin alpha 1B (Uniprot ID P81947) and beta-2B (Q6B856) chains with the known inhibitors, namely, colchicine and combretastatin, were downloaded from Protein DataBank (https://www.rcsb.org/). Water molecules and native inhibitors were removed from the structures and 3D structure of the unresolved residues was rebuilt by Modeller Tool^15^ built-in Chimera^16^. Additional refinements, such as remodelling of incomplete side chains and protonation of the protein structure were performed by Chimera Dock Prep tool^16^. Notably, the cofactors like GTP and Mg^2+^ ions, which are important for polymerisation regulation, were kept.

#### Molecular docking

The docking procedure employed AutoDock Vina^17^ to dock all compounds. Given the substantial size of the ED molecules, a docking box of dimensions 28 × 28 × 28 Å was utilised, centred around the active site. To balance accuracy and efficiency, the exhaustiveness value was set to 32. Initial conformers were generated using RDKit version 2018.09.1.0^18^. For ligand protonation at pH 7.4, the Marvin cxcalc utility was employed^19^.

#### Molecular dynamics

Simulations were performed using GROMACS software version 2018.1-intel-2017c-hybrid-single-PLUMED^20^. For target preparation, we used the Amber 99SB-ILDN force field^21^ and the TIP3P water model. Na and Cl ions were added to neutralise the system. Ligand topologies were prepared by AmberTools version 20.9^22^. Energy minimisation for every simulation took 50 000 steps, followed by NVT and then NPT equilibrations for 1000 ps. Production simulations were conducted for 150 ns in an NPT ensemble at 300 K. For the visualisation and analysis of the protein–ligand interaction, we used the ProLIF package^11^, using only frames extracted from the last 100 ns^10^.

## Supplementary Material

Supplemental Material

## Data Availability

The datasets presented in this study are available from the corresponding author upon reasonable request.
