# Supplementary material for: Click estradiol dimers with novel aromatic bridging units: synthesis and anticancer evaluation
Source: J Enzyme Inhib Med Chem. 2024 Jun 21;39(1):2367139. doi: 10.1080/14756366.2024.2367139 (PMC467089; doi:10.1080/14756366.2024.2367139)
Supplement: Supplemental Material [file IENZ_A_2367139_SM5172.pdf]

Supplementary Material

Click Estradiol Dimers with Novel Aromatic Bridging Units: Synthesis and Anticancer Evaluation

Jiří Řehulka[a], Michal Jurášek[b]*, Pavel Dráber[c], Aleksandra Ivanová[a], Soňa Gurská[a], Kateřina Ječmeňová[a], Olena Mokshyna[a,d], Marián Hajdúch[a,e], Pavel Polishchuk[a], Pavel B. Drašar[b] and Petr Džubák[a,e]*

[a] *Institute of Molecular and Translational Medicine, Faculty of Medicine and Dentistry, Palacký University, Hněvotínská 976/3, 779 00 Olomouc, Czech Republic*
[b] *Department of Chemistry of Natural Compounds, University of Chemistry and Technology Prague, Technická 5, 166 28 Praha 6, Czech Republic*
[c] *Institute of Molecular Genetics of the Czech Academy of Sciences, Department of Biology of Cytoskeleton, Vídeňská 1083, 142 20 Praha 4, Czech Republic*
[d] *Institute of Organic Chemistry and Biochemistry of the Czech Academy of Sciences, Flemingovo náměstí 542/2, 160 00 Praha 6, Czech Republic*
[e] *Laboratory of Experimental Medicine, Institute of Molecular and Translational Medicine, University Hospital Olomouc, Hněvotínská 976/3, 77900 Olomouc, Czech Republic*

1. Analytical spectra

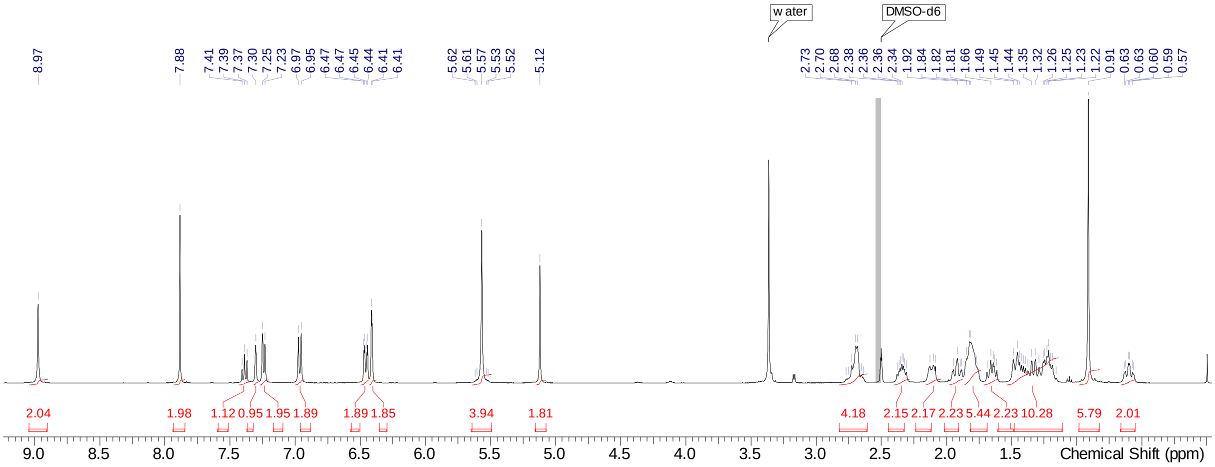

Figure S1. ^{1}H NMR of **ED1** in DMSO-*d6*

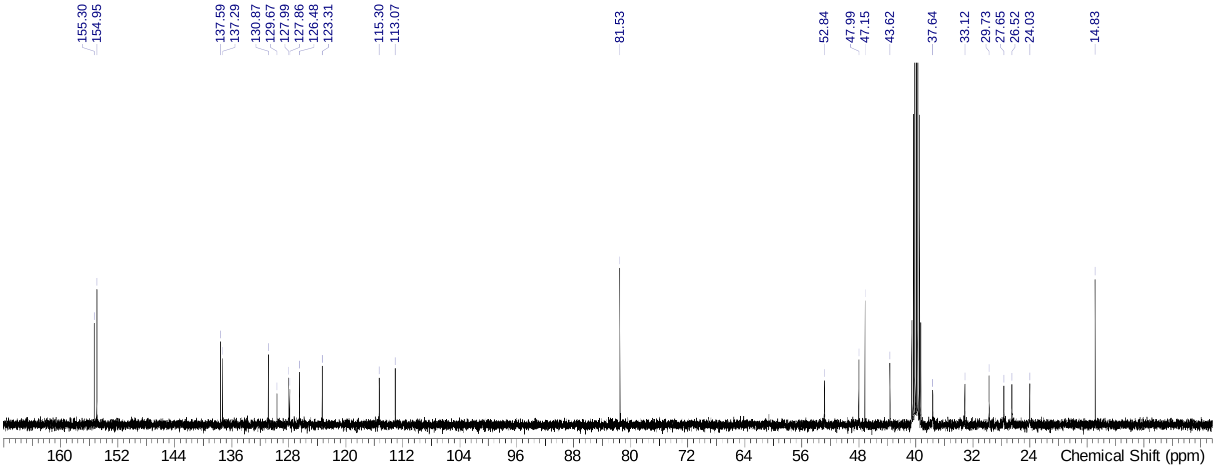

Figure S2. ^{13}C NMR of **ED1** in DMSO-*d6*

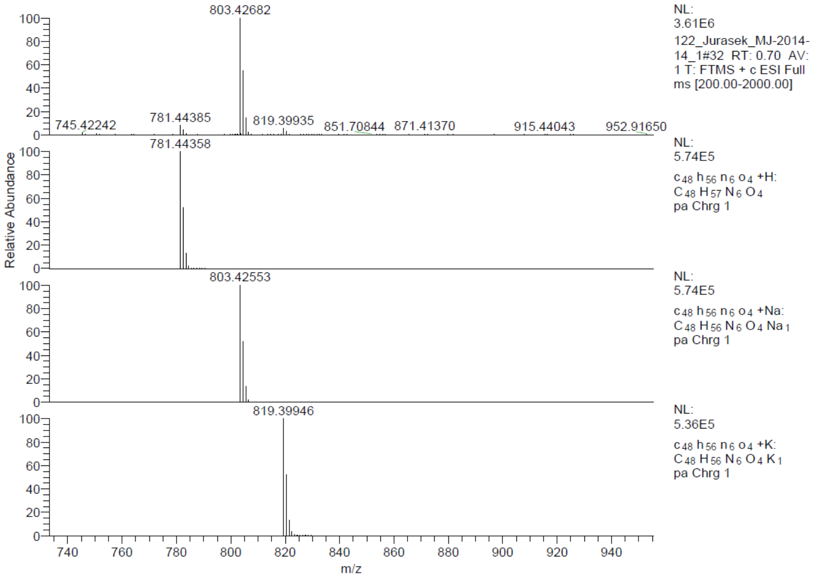

Figure S3. HRMS-ESI of **ED1**

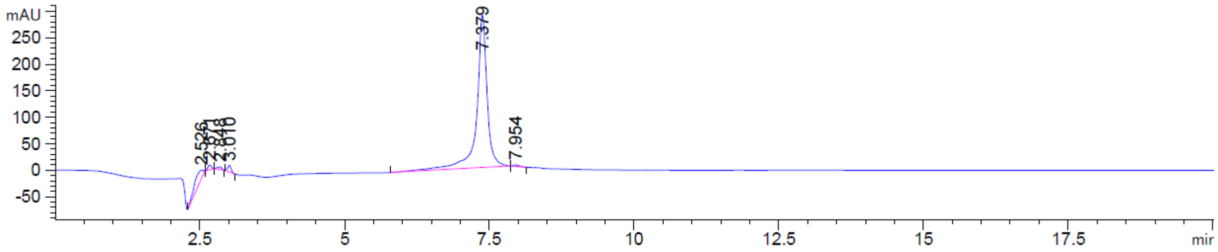

Figure S4. HPLC of **ED1**

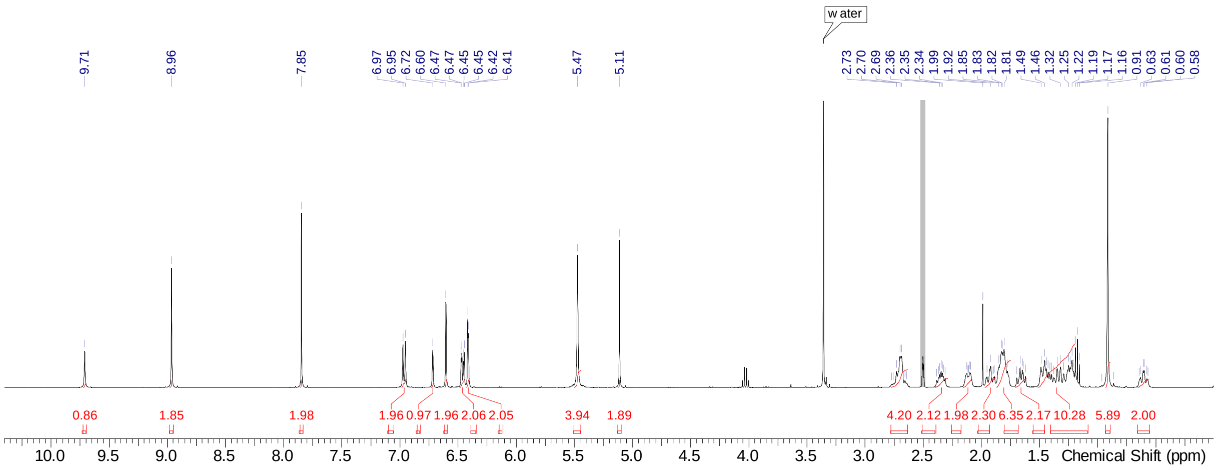

Figure S5. ¹H NMR of **ED2** in DMSO-*d6*

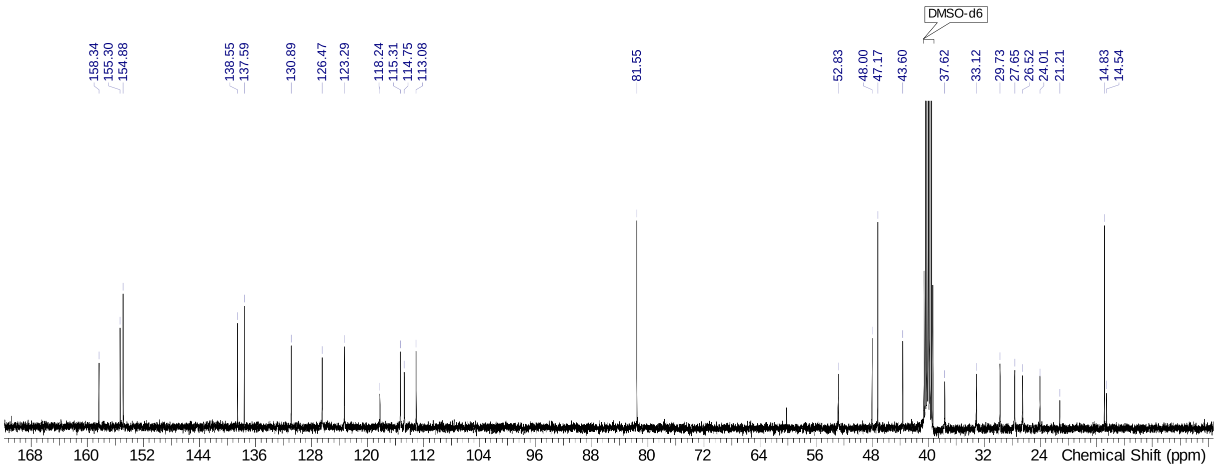

Figure S6. ¹³C NMR of **ED2** in DMSO-*d6*

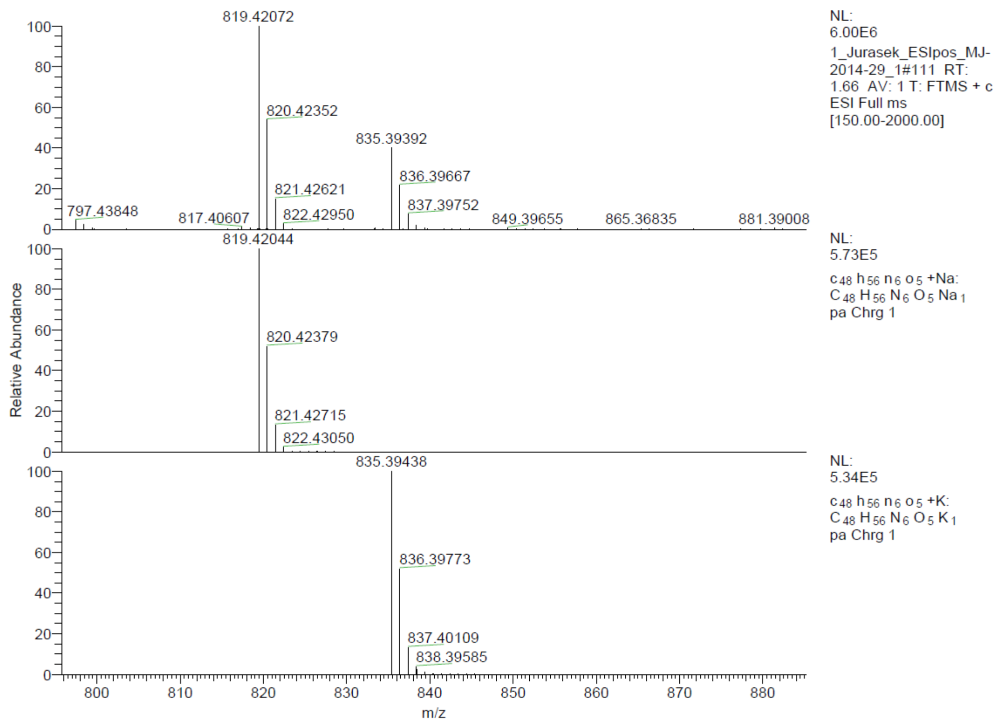

Figure S7. HRMS-ESI of **ED2**

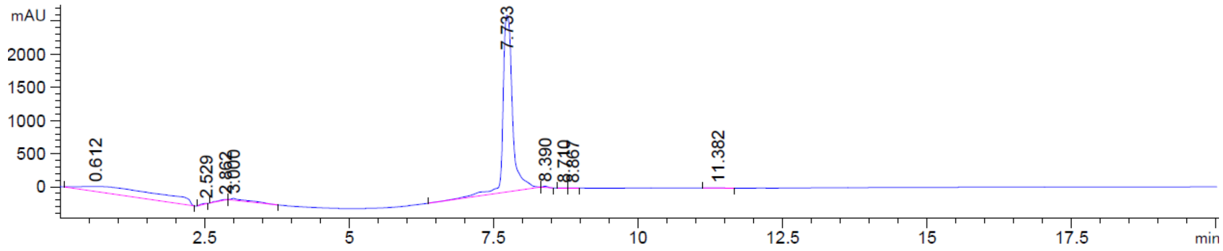

Figure S8. HPLC of **ED2**

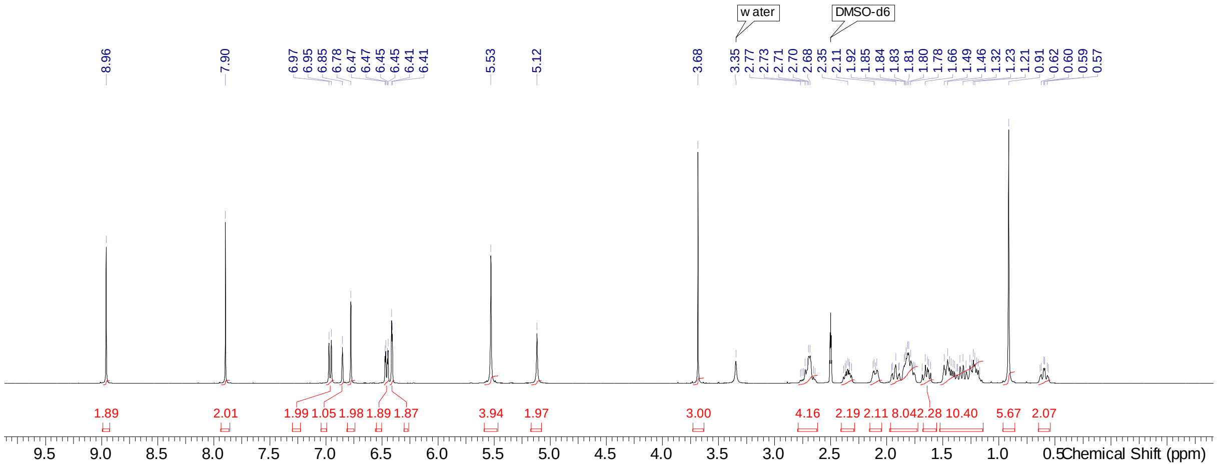

Figure S9. ^{1}H NMR of **ED3** in DMSO-*d6*

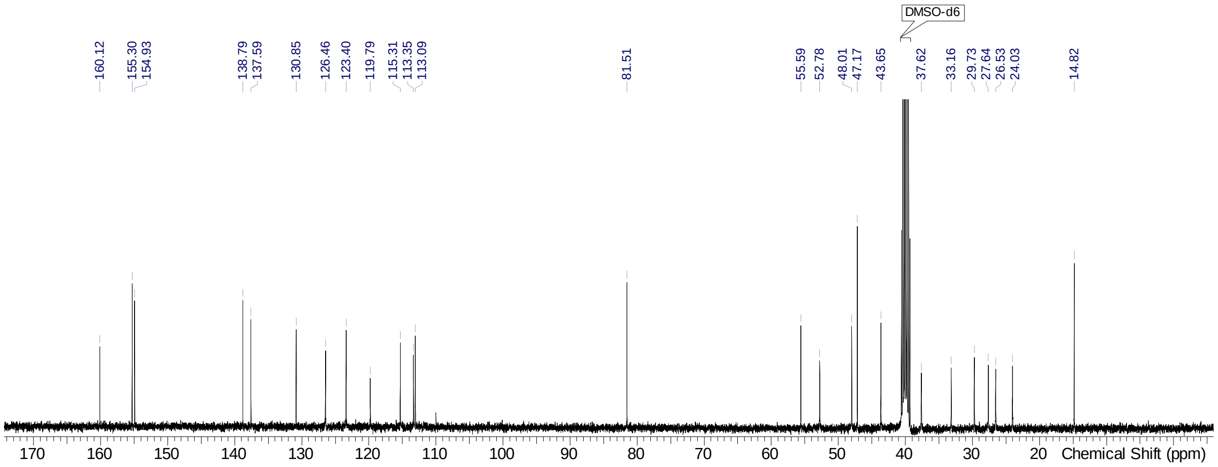

Figure S10. ^{13}C NMR of **ED3** in DMSO-*d6*

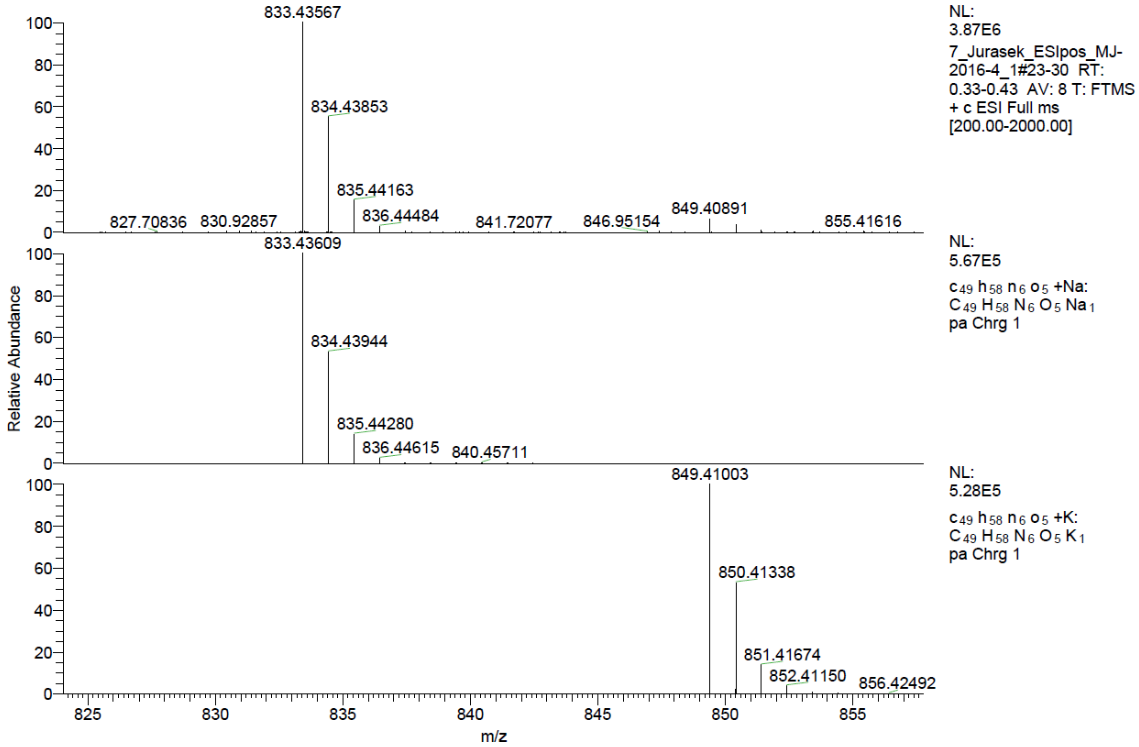

Figure S11. HRMS-ESI of **ED3**

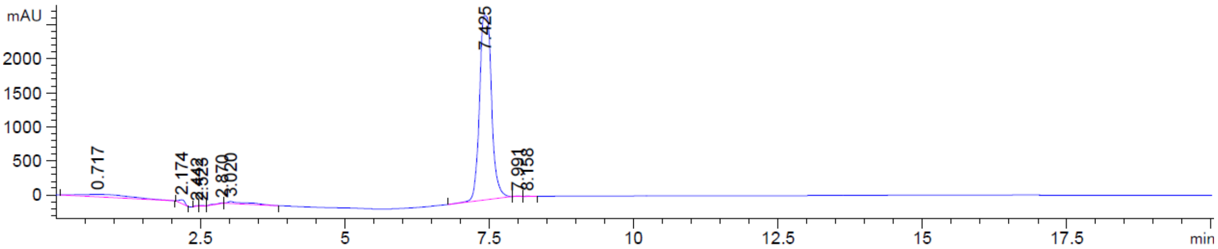

Figure S12. HPLC of **ED3**

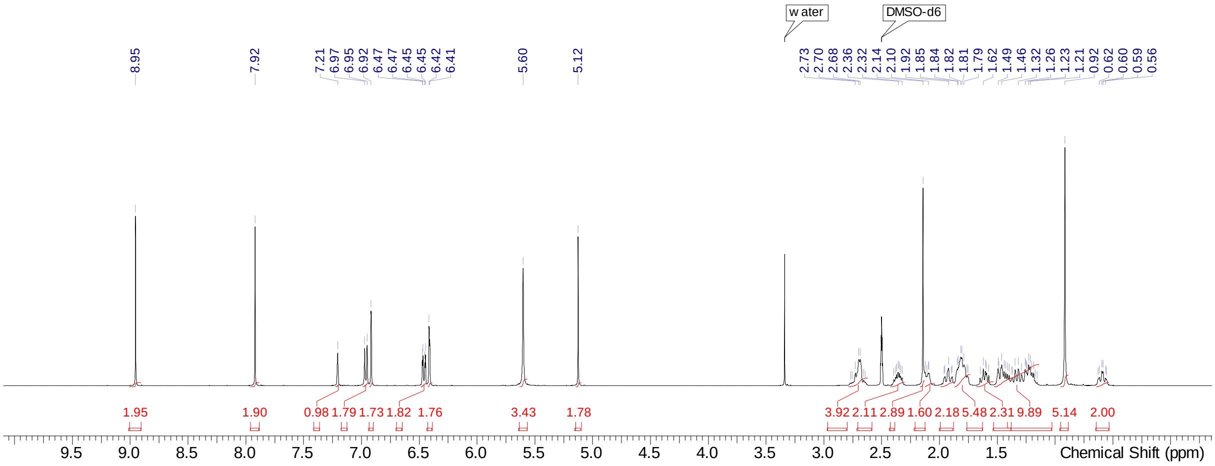

Figure S13. ^{1}H NMR of **ED4** in DMSO-*d6*

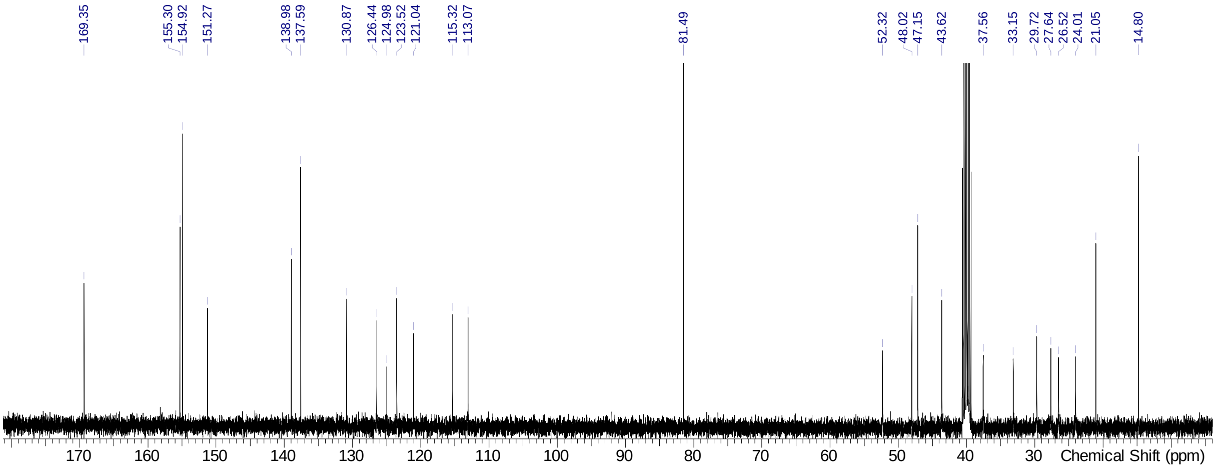

Figure S14. ^{13}C NMR of **ED4** in DMSO-*d6*

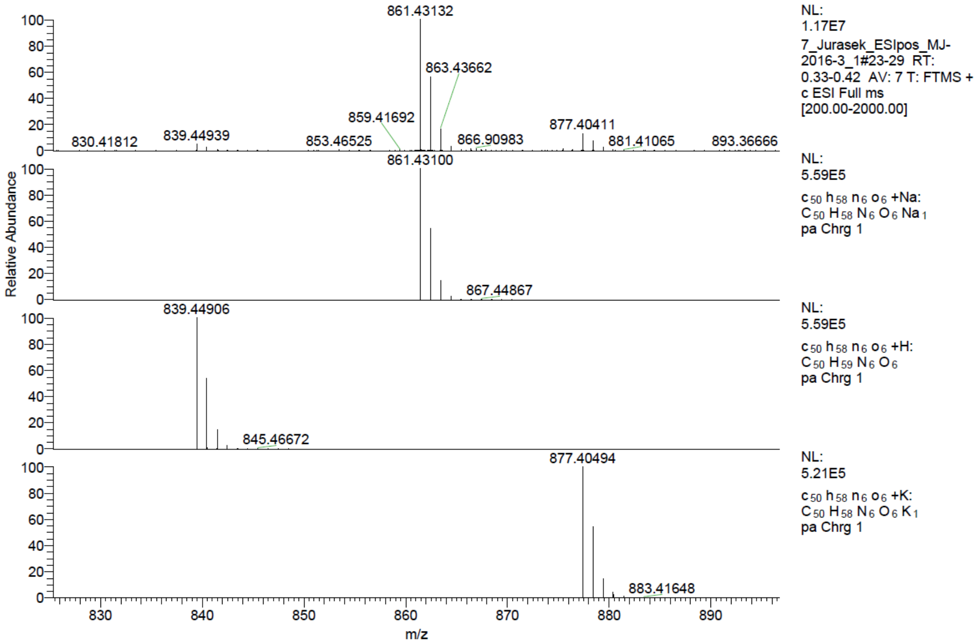

Figure S15. HRMS-ESI of **ED4**

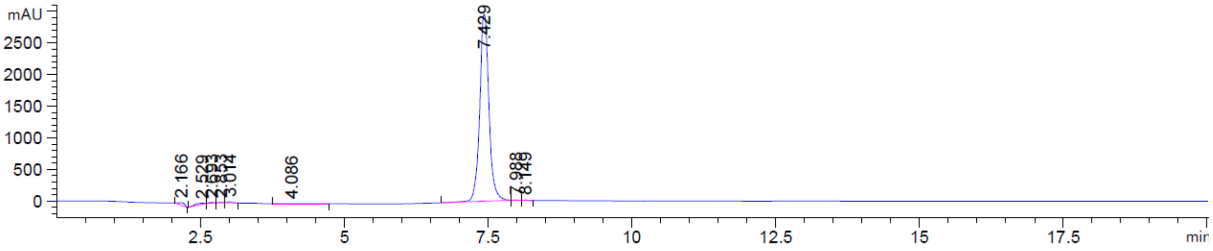

Figure S16. HPLC of **ED4**

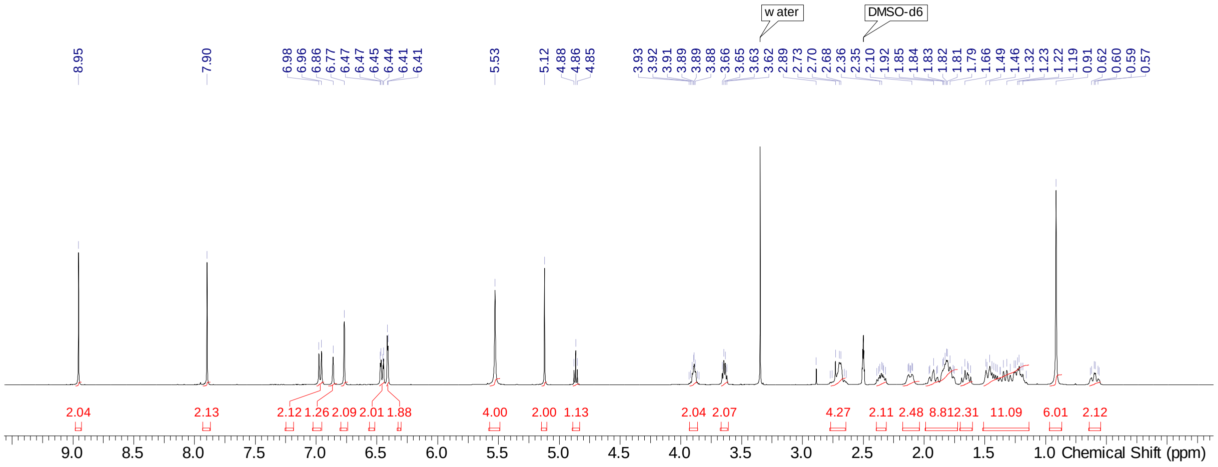

Figure S17. ^{1}H NMR of **ED5** in DMSO-*d6*

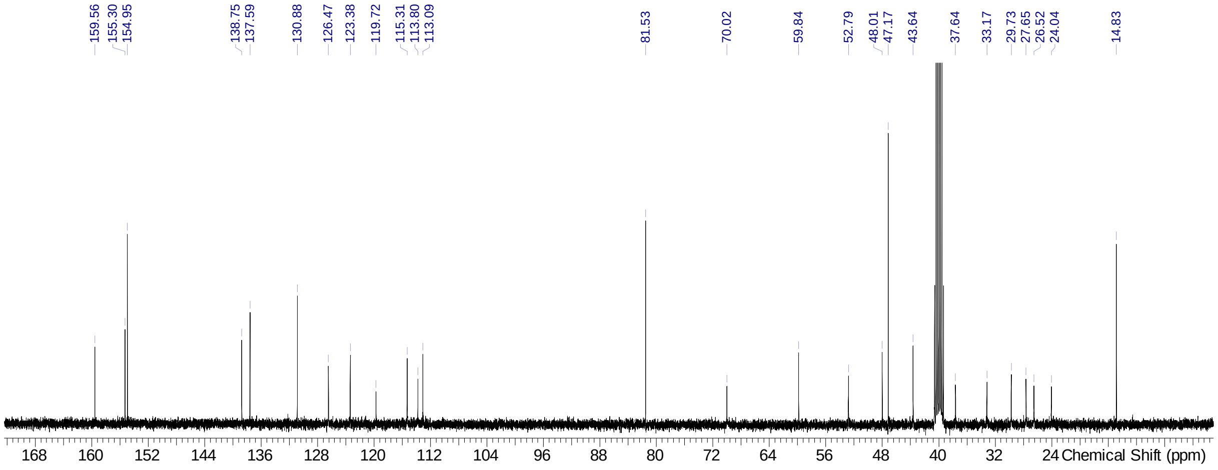

Figure S18. ^{13}C NMR of **ED5** in DMSO-*d6*

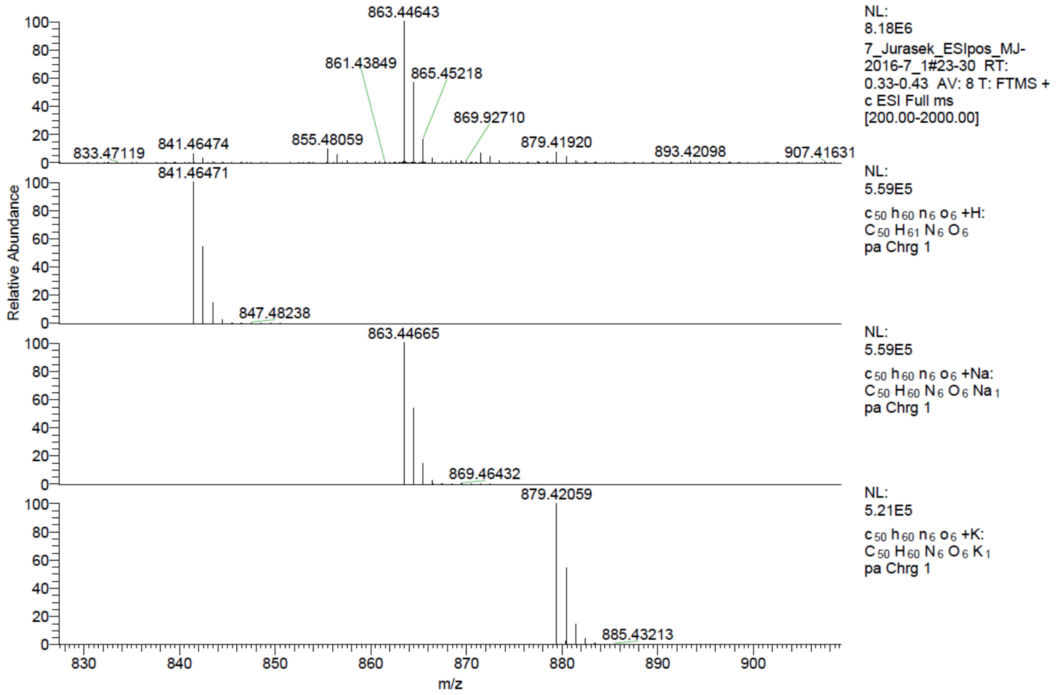

Figure S19. HRMS-ESI of **ED5**

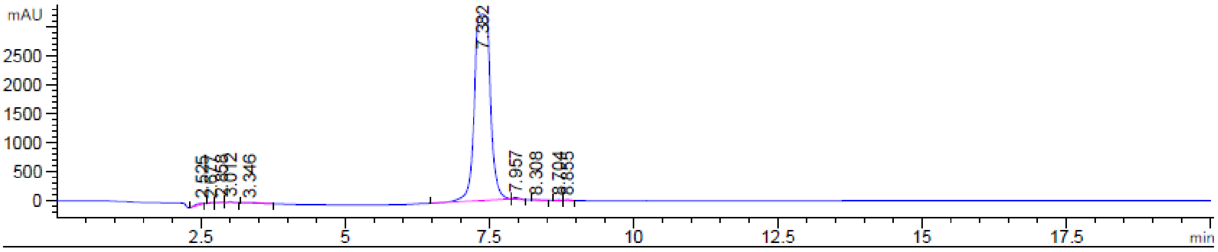

Figure S20. HPLC of **ED5**

- <u>Analyses of **ED6**</u>

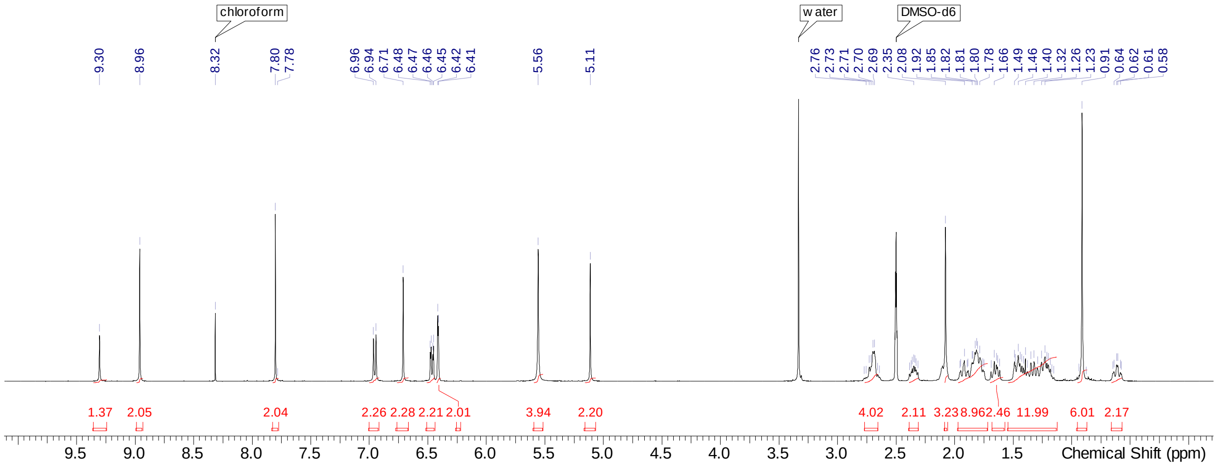

Figure S21. ^{1}H NMR of **ED6** in DMSO-*d6*

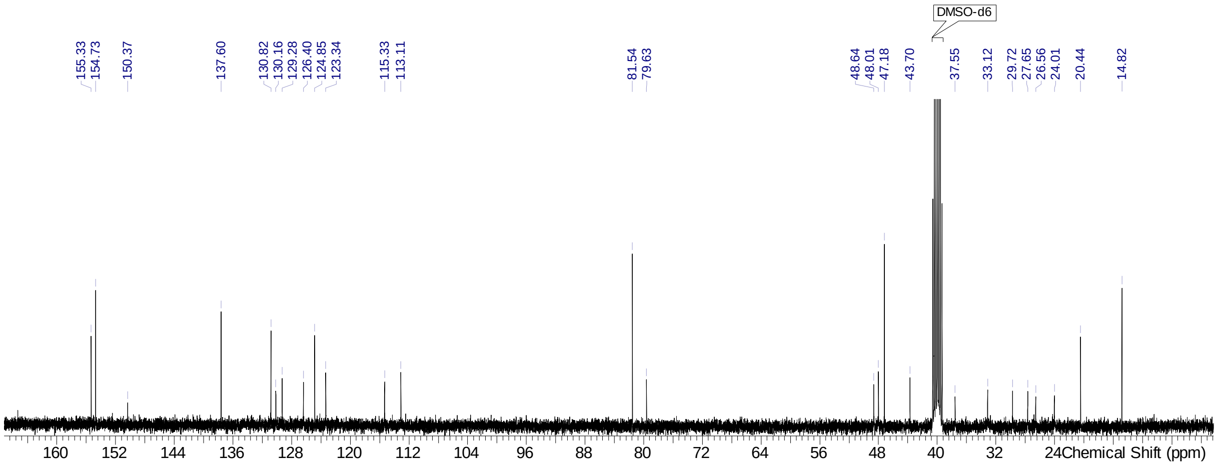

Figure S22. ^{13}C NMR of **ED6** in DMSO-*d6*

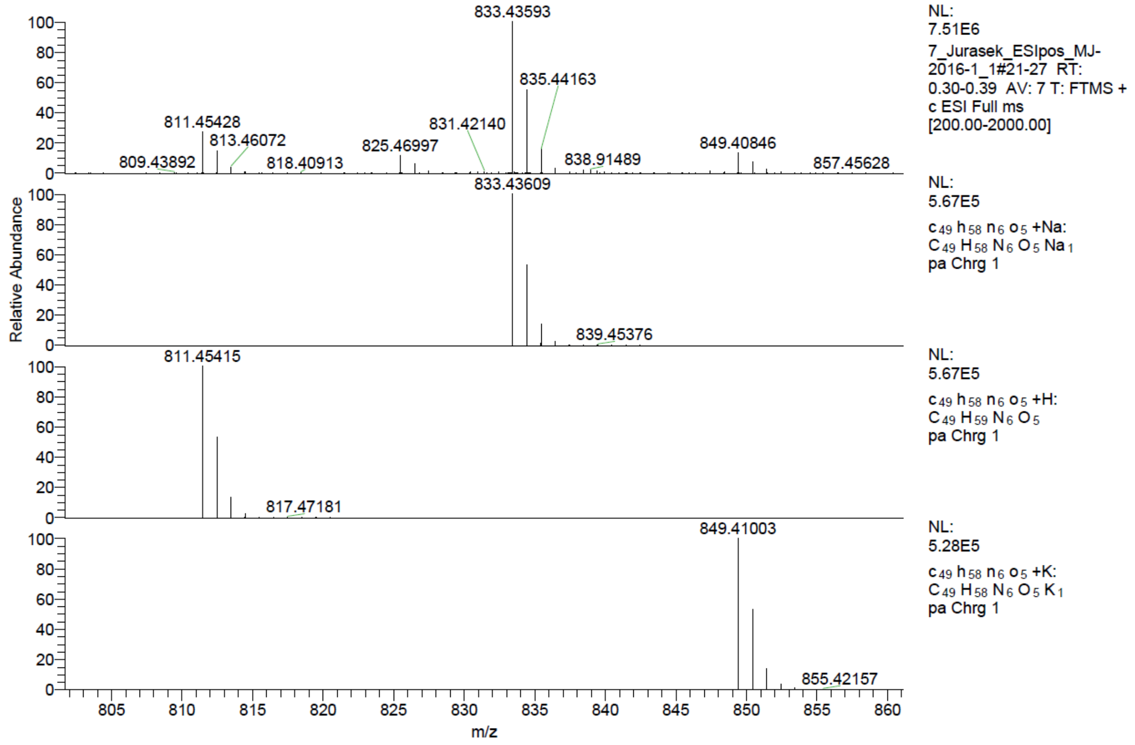

Figure S23. HRMS-ESI of **ED6**

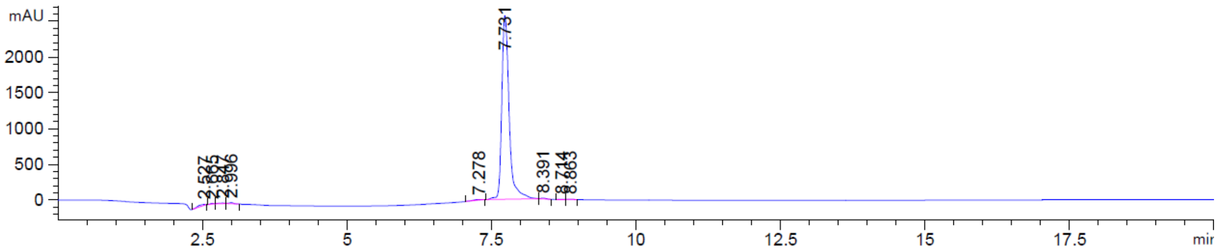

Figure S24. HPLC of **ED6**

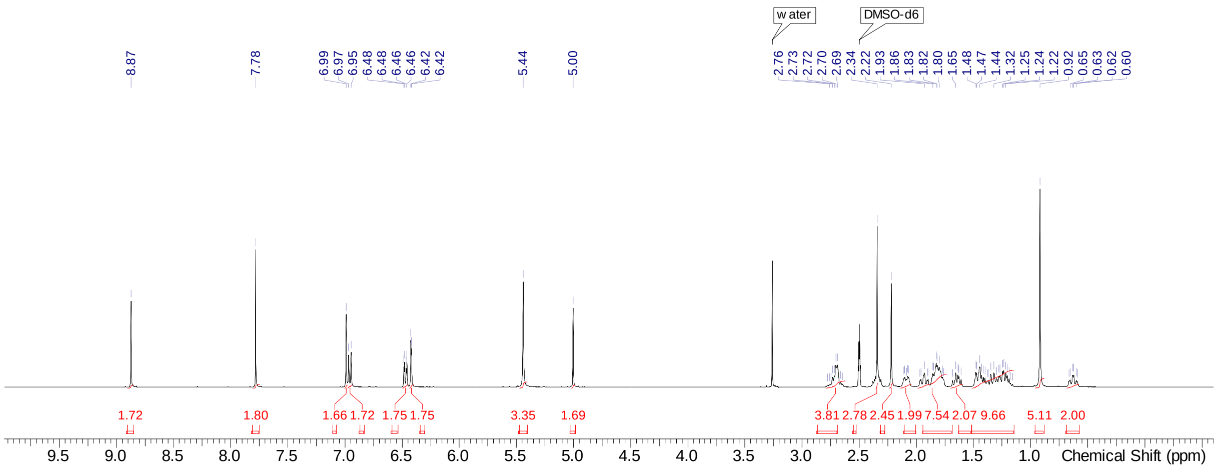

Figure S25. ^{1}H NMR of **ED7** in DMSO-*d6*

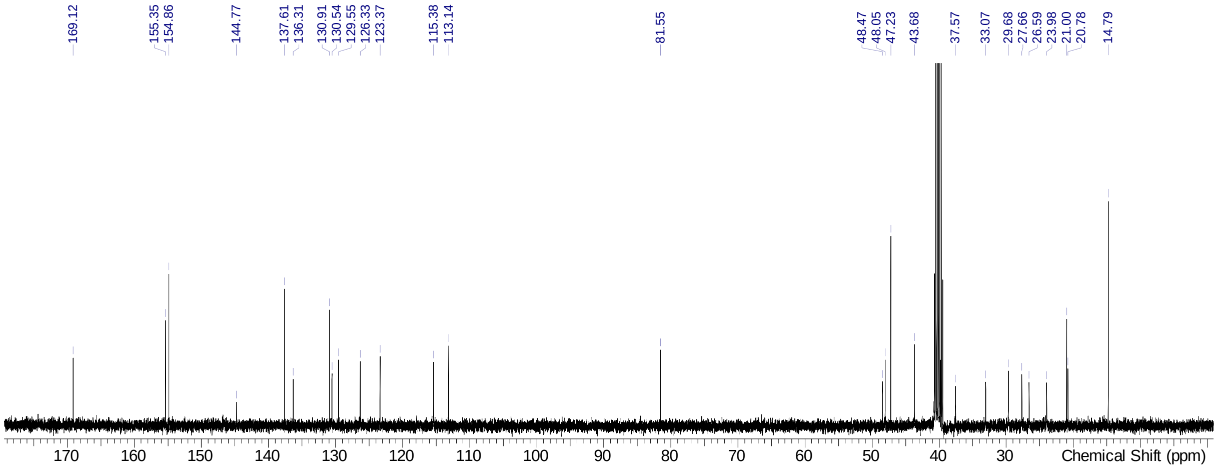

Figure S26. ^{13}C NMR of **ED7** in DMSO-*d6*

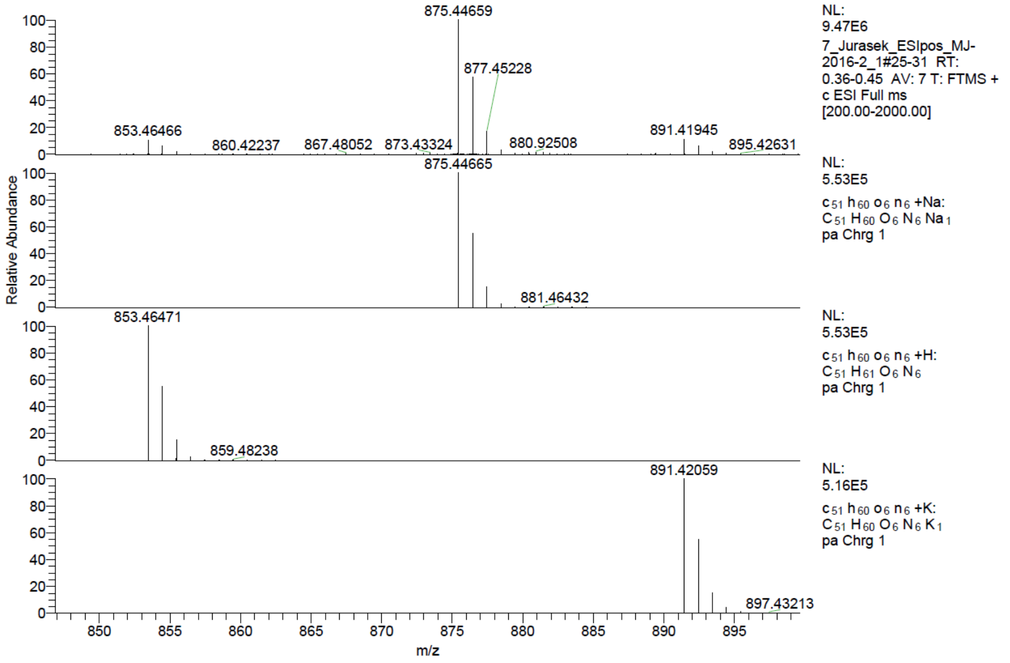

Figure S27. HRMS-ESI of **ED7**

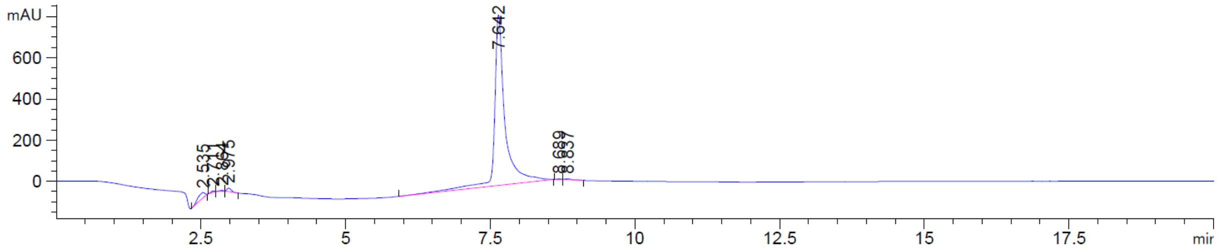

Figure S28. HPLC of **ED7**

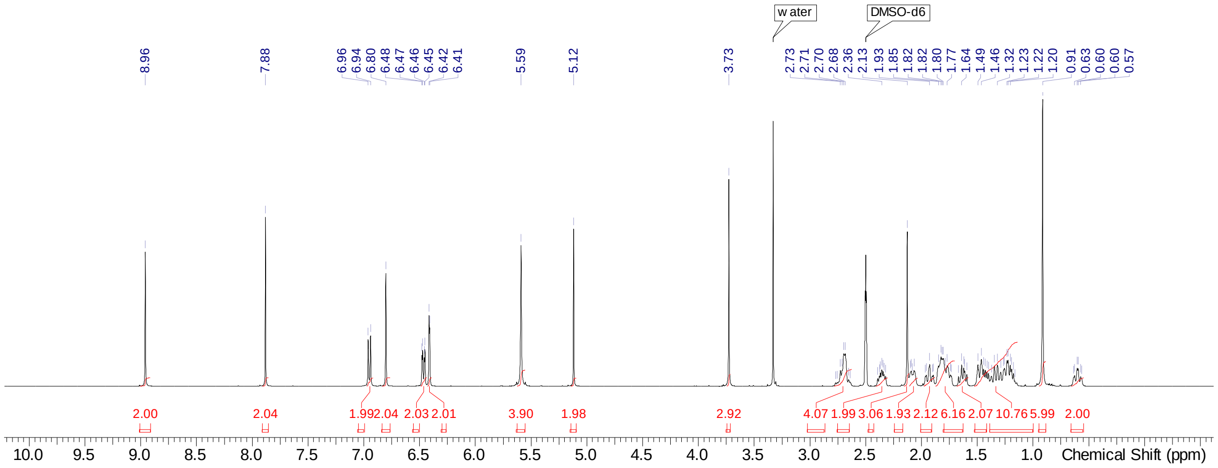

Figure S29. ^{1}H NMR of **ED8** in DMSO-*d6*

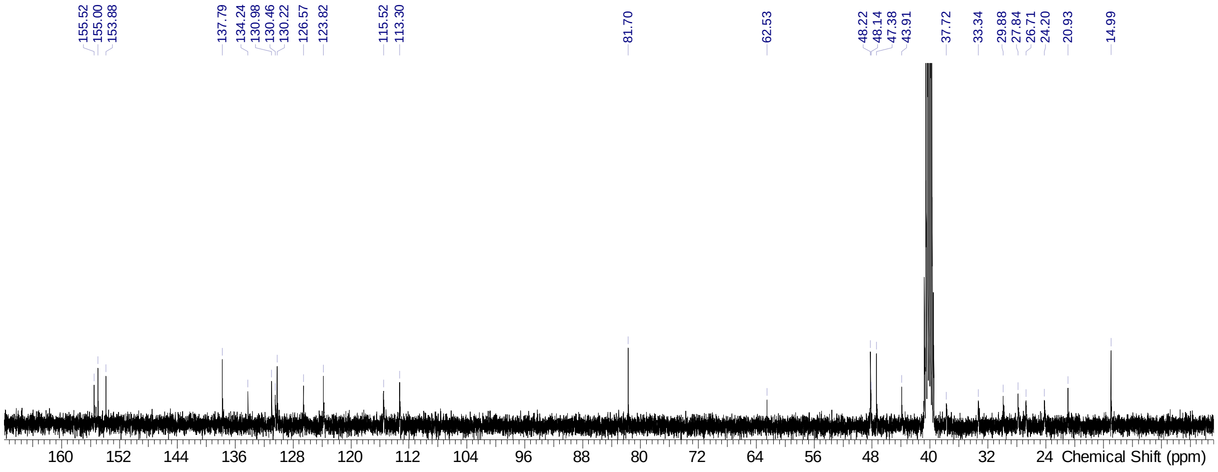

Figure S30. ^{13}C NMR of **ED8** in DMSO-*d6*

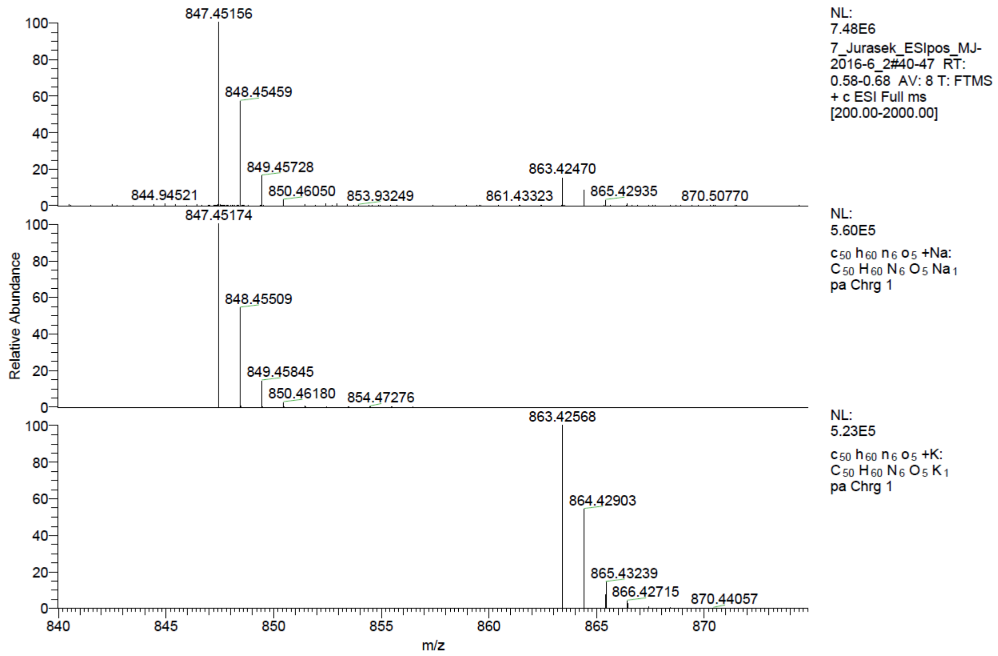

Figure S31. HRMS-ESI of **ED8**

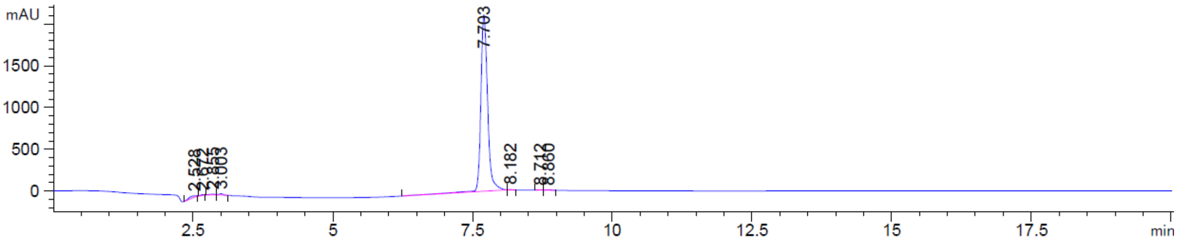

Figure S32. HPLC of **ED8**

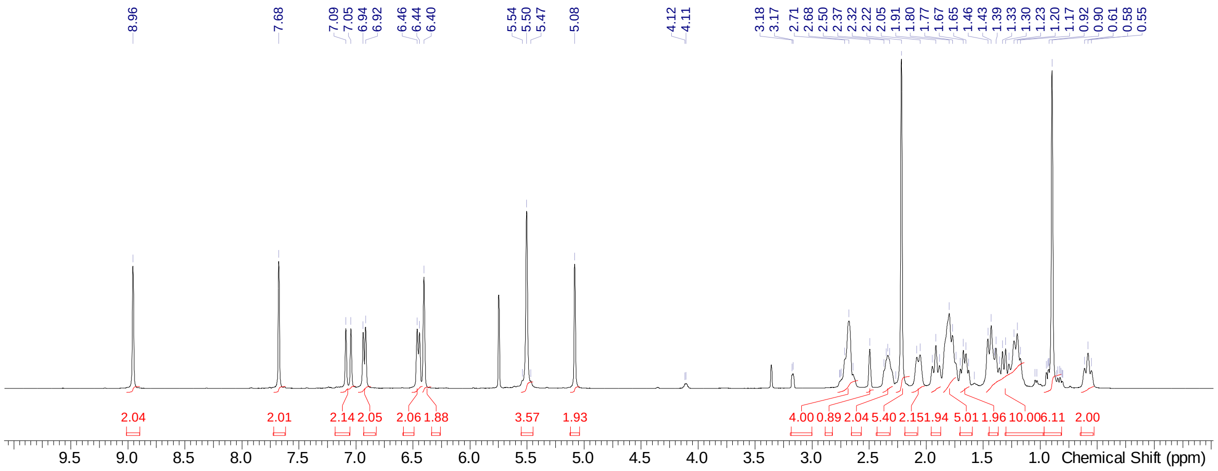

Figure S33. ^{1}H NMR of **ED9** in DMSO-*d6*

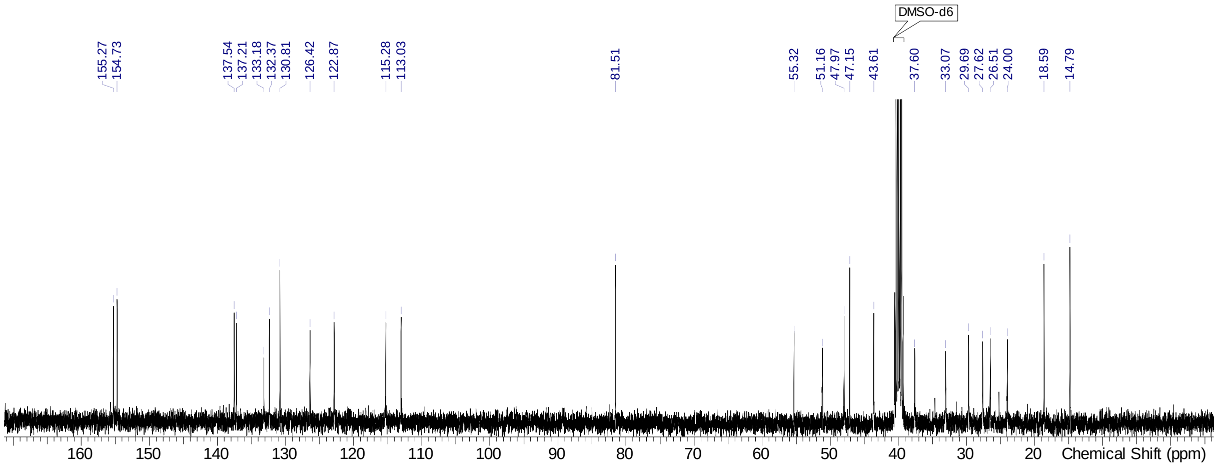

Figure S34. ^{13}C NMR of **ED9** in DMSO-*d6*

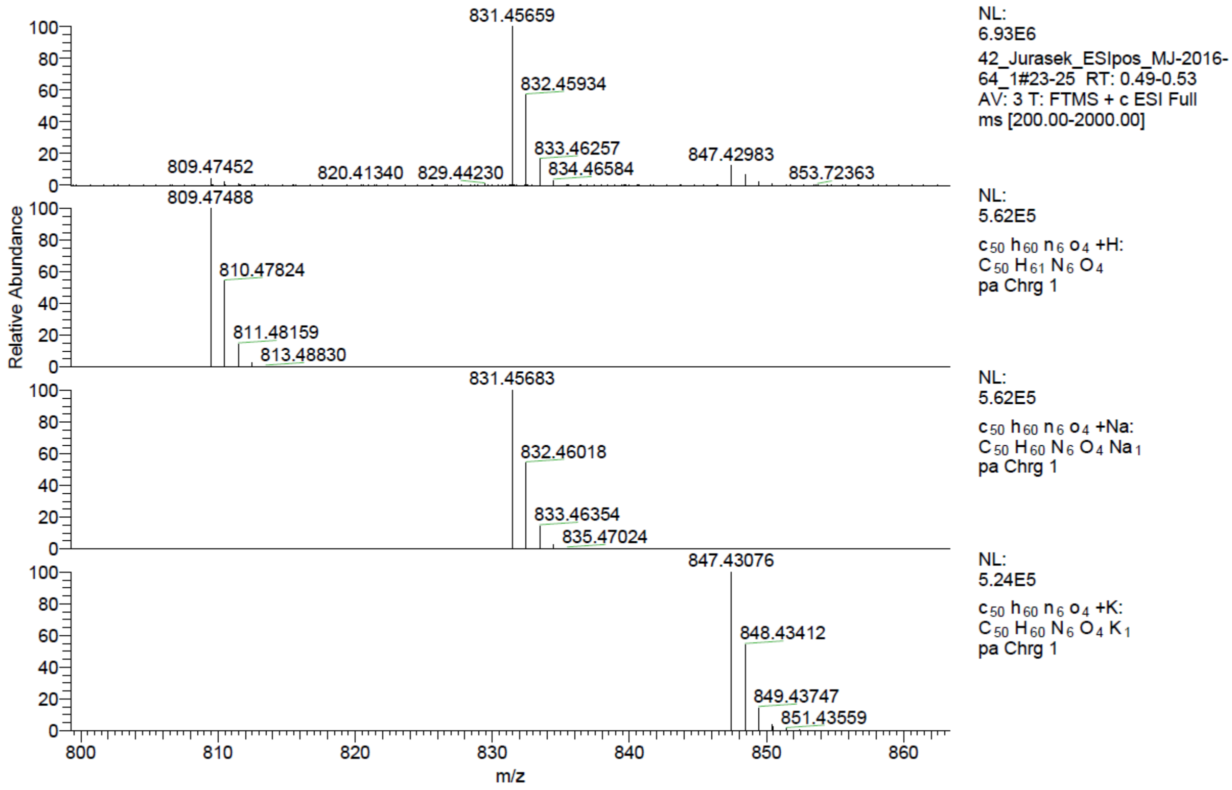

Figure S35. HRMS-ESI of **ED9**

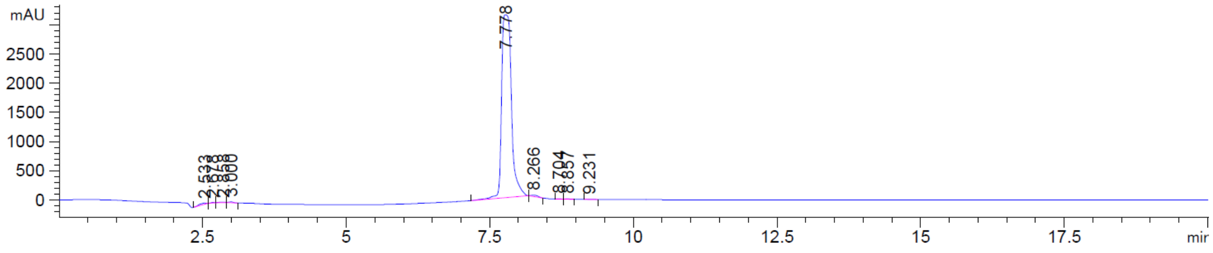

Figure S36. HPLC of **ED9** (mobile system A)

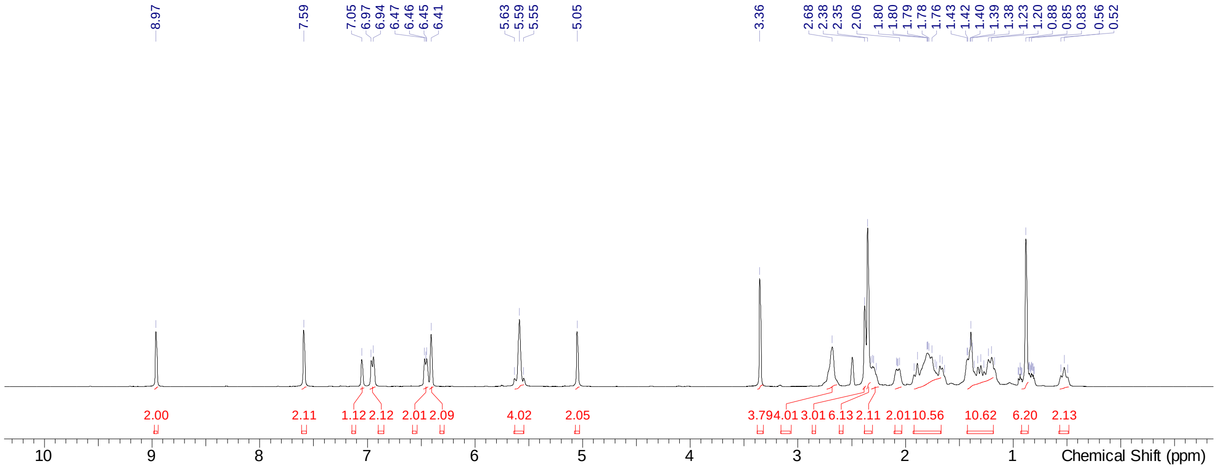

Figure S37. ^{1}H NMR of **ED10** in DMSO-*d6*

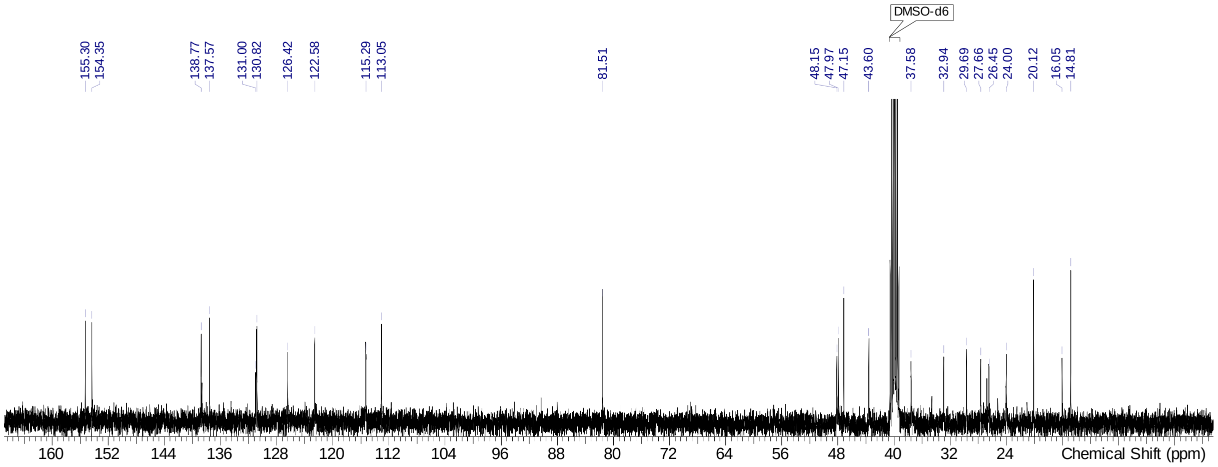

Figure S38. ^{13}C NMR of **ED10** in DMSO-*d6*

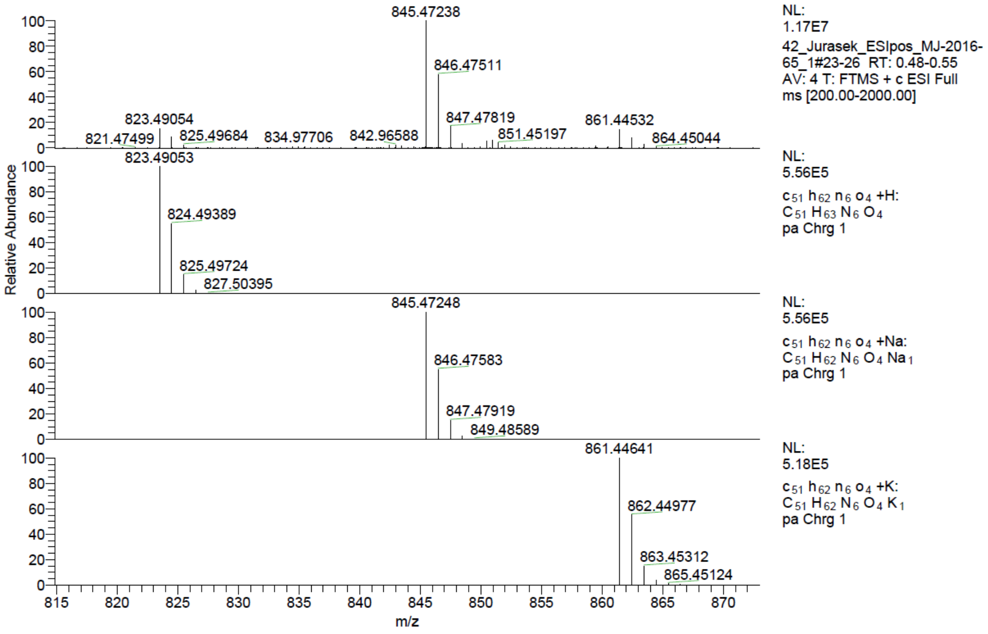

Figure S39. HRMS-ESI of **ED10**

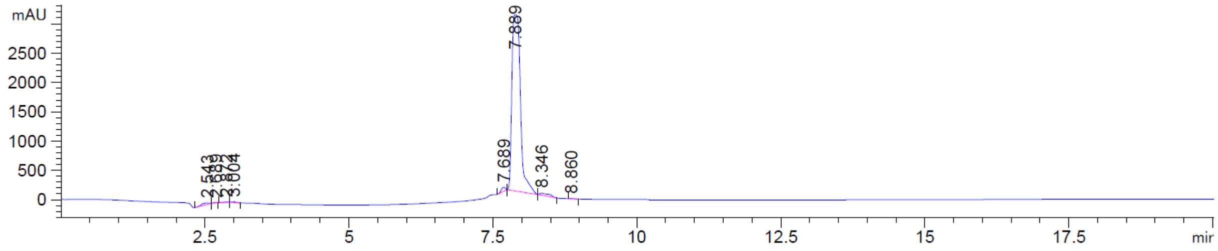

Figure S40. HPLC of **ED10**

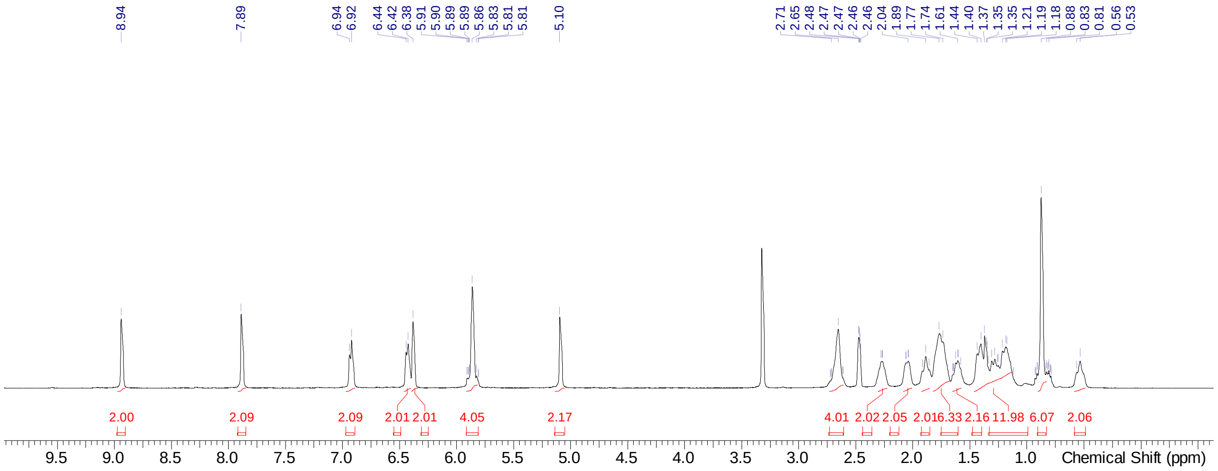

Figure S41. ^{1}H NMR of **ED11** in DMSO-*d6*

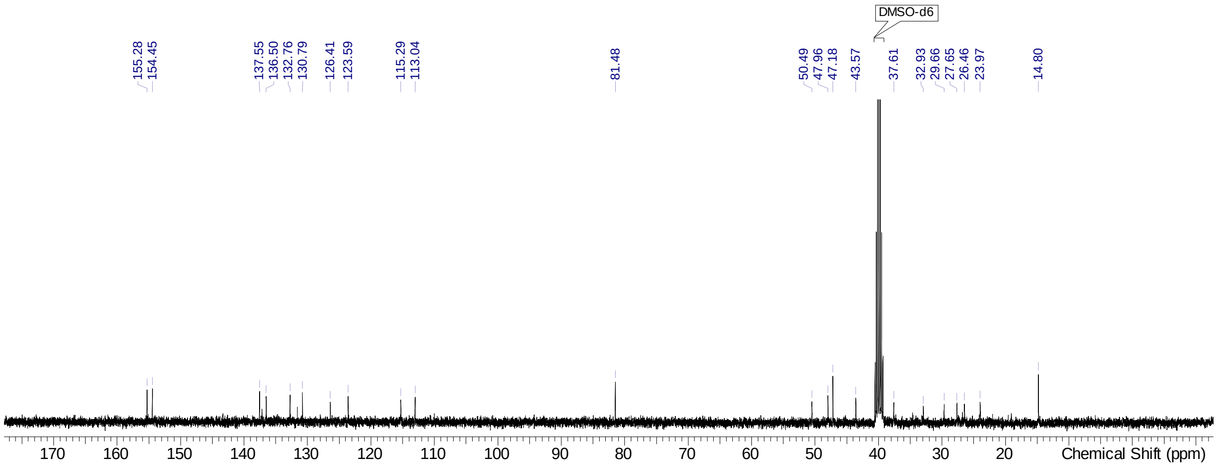

Figure S42. ^{13}C NMR of **ED11** in DMSO-*d6*

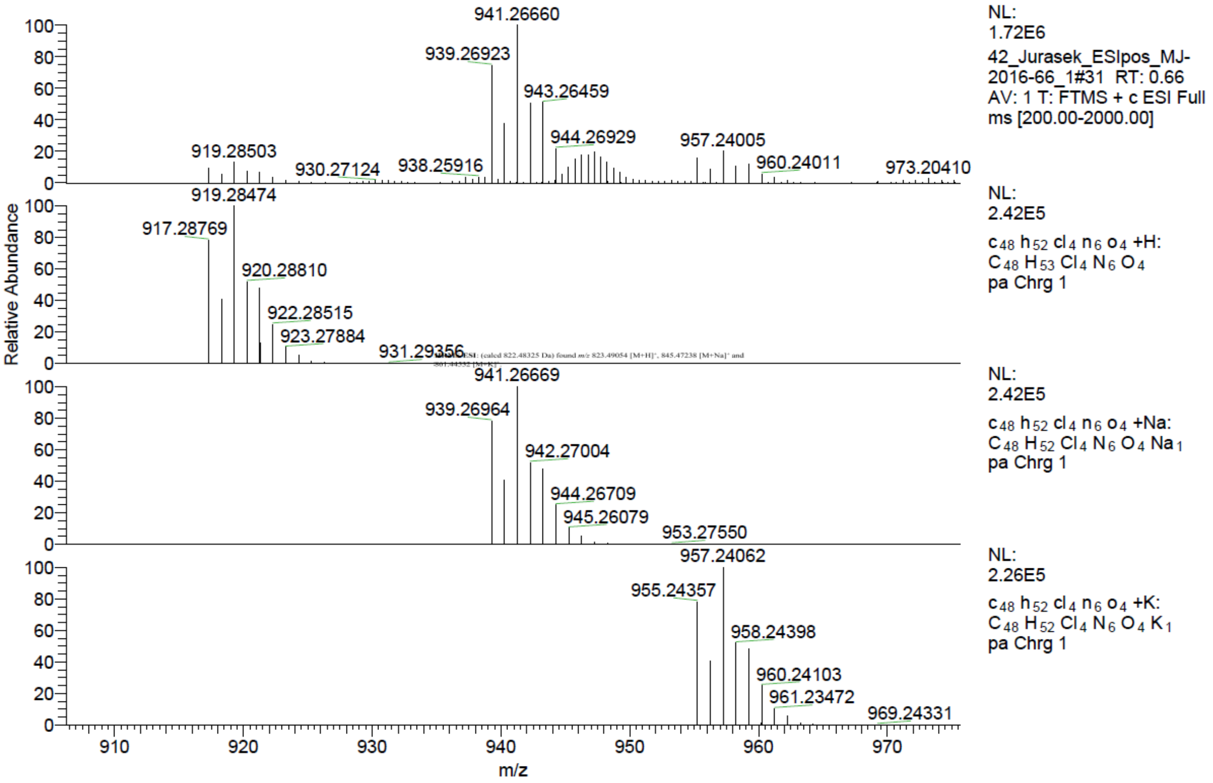

Figure S43. HRMS-ESI of **ED11**

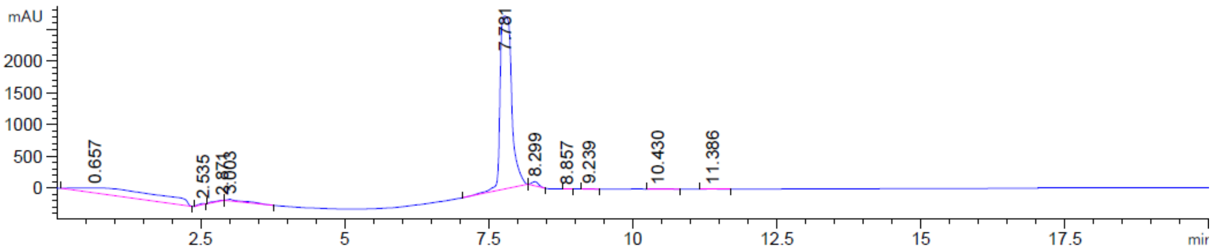

Figure S44. HPLC of **ED11**

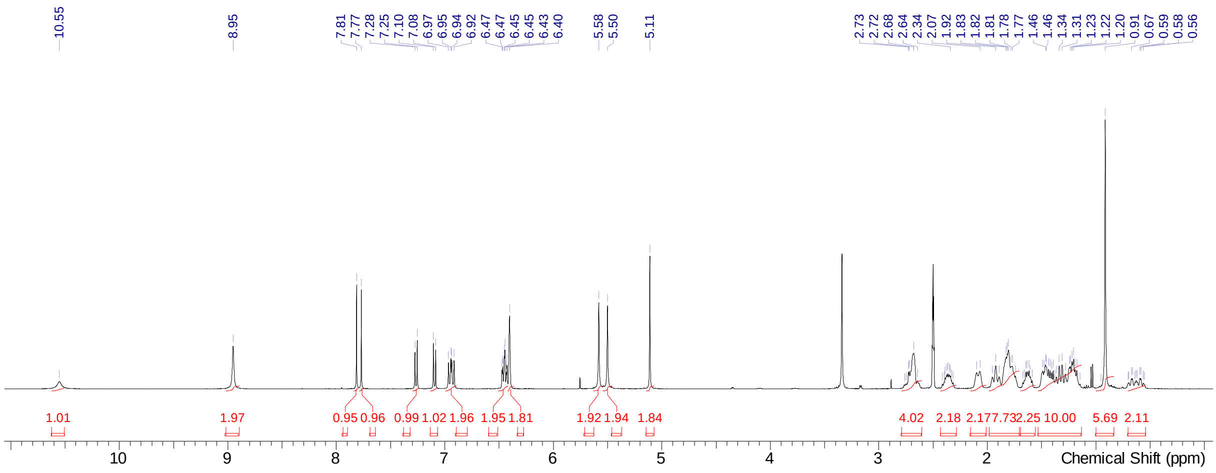

Figure S45. ^{1}H NMR of **ED12** in DMSO-*d6*

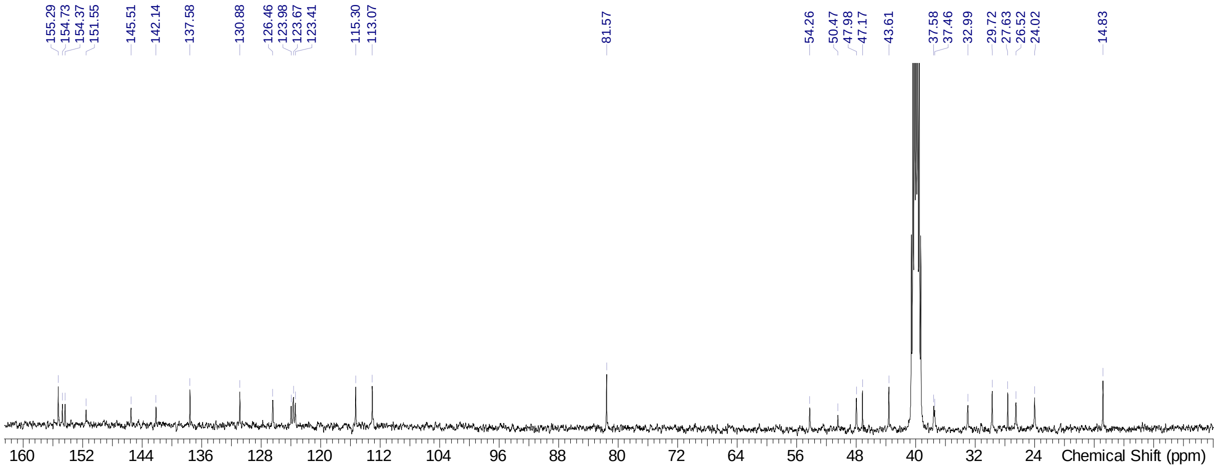

Figure S46. ^{13}C NMR of **ED12** in DMSO-*d6*

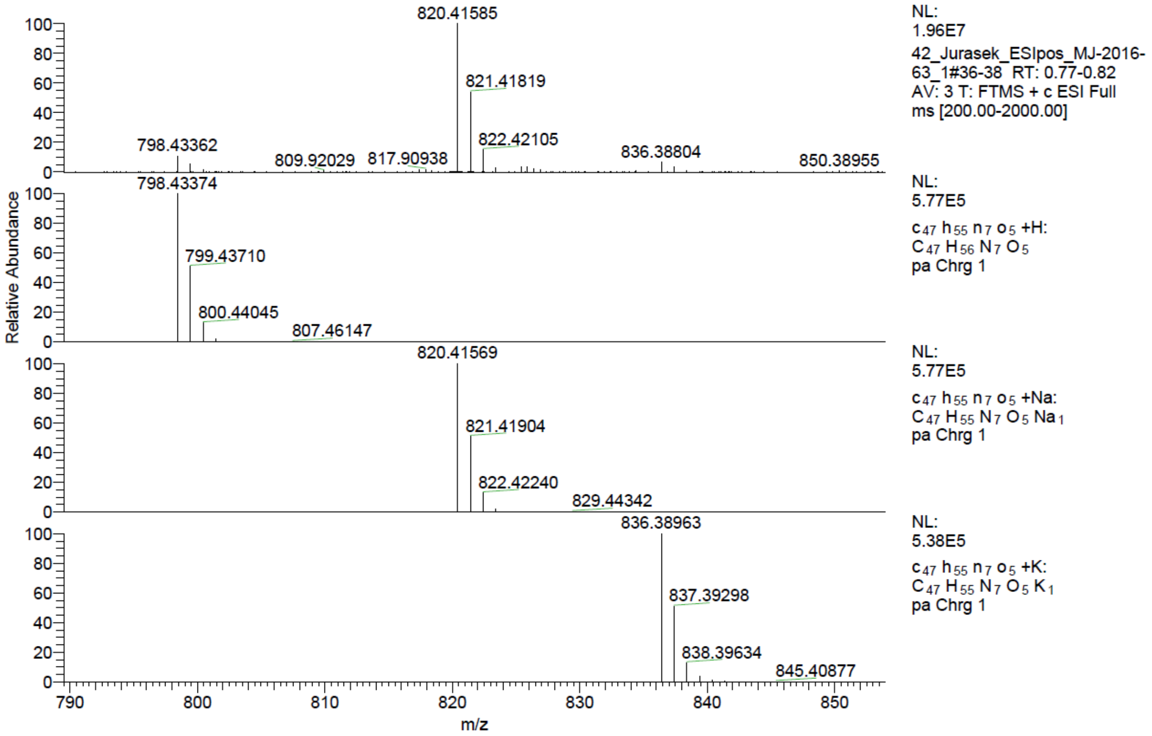

Figure S47. HRMS-ESI of **ED12**

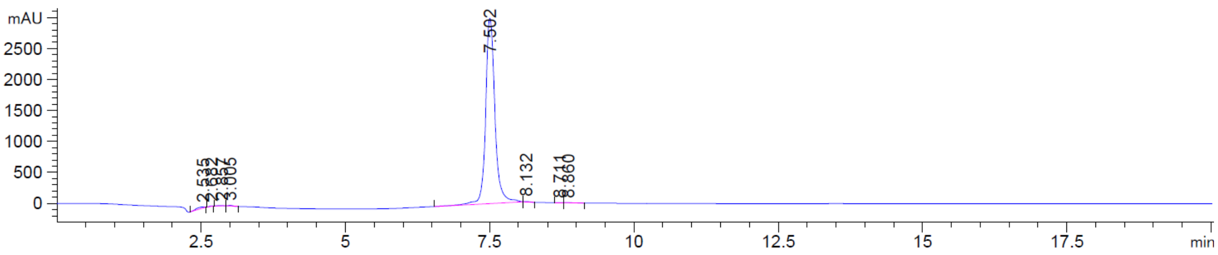

Figure S48. HPLC of **ED12** (mobile system A)

2. Biochemistry

Table S1. Effect of cytotoxic compounds on cell cycle, induction of apoptosis and polyploidy and DNA/RNA synthesis in CCRF-CEM lymphoblasts (% of positive cells); Flow cytometry analysis was used for quantification of cell cycle distribution and apoptotic cells. [a]phospho-Histone (Ser10); [b]5-bromo-2-deoxyuridine; [c]BrU, 5-bromouridine.

**Compound**	**<G1**	**G_0/G_1**	**S**	**G2/M**	**$>G_2/M$**	**pH3Ser[10a]**	**BrDU[b]**	**BrU[c]**
control	5.33	40.95	41.35	17.70	6.9	1.49	43.40	47.61
**ME** 1×IC_{50}	23.97	31.50	39.65	28.85	6.2	17.74	9.31	23.60
**ME** 5×IC_{50}	65.32	1.19	14.77	84.03	10.4	67.40	9.49	46.10
**ED** 1×IC_{50}	11.51	33.48	40.93	25.59	11.2	8.08	19.54	34.33
**ED** 5×IC_{50}	22.78	2.50	38.39	59.11	23.1	48.71	15.94	15.43
**ED1** 1×IC_{50}	55.53	0.28	25.01	74.70	10.7	61.09	38.15	48.16
**ED1** 5×IC_{50}	64.75	0.27	24.67	75.07	17.65	64.45	6.70	31.42
**ED2** ×IC_{50}	28.21	2.32	32.78	64.90	8.33	52.73	15.09	41.11
**ED2** 5×IC_{50}	19.20	0.47	41.13	58.40	8.13	47.87	21.14	41.82
**ED3** 1×IC_{50}	33.51	6.68	22.67	70.65	10.23	1.43	7.81	68.66
**ED3** 5×IC_{50}	53.18	6.89	21.44	71.67	9.8	0.77	3.23	60.00
**ED4** 1×IC_{50}	61.81	25.61	33.89	40.50	6.75	1.25	26.50	66.58
**ED4** 5×IC_{50}	31.27	8.75	19.84	71.41	10.24	0.12	7.16	3.26
**ED5** 1×IC_{50}	29.35	35.53	37.57	26.90	6.16	6.27	32.15	65.14
**ED5** 5×IC_{50}	36.87	13.42	25.09	61.48	10.73	0.34	9.59	66.40
**ED6** 1×IC_{50}	11.43	32.46	37.81	29.74	6.19	9.07	37.25	67.97
**ED6** 5×IC_{50}	36.87	8.21	18.50	73.29	9.85	0.76	7.83	39.43
**ED7** 1×IC_{50}	40.74	32.36	33.88	33.76	7.12	0.68	9.50	52.67
**ED7** 5×IC_{50}	45.33	32.48	34.65	32.87	7.43	11.22	5.65	27.20
**ED8** 1×IC_{50}	16.29	34.59	33.47	31.94	7.25	11.01	45.06	59.19
**ED8** 5×IC_{50}	24.86	4.71	16.36	78.93	10.51	0.63	5.15	3.11
**ED9** 1×IC_{50}	3.92	38.18	37.90	23.92	13.86	8.42	46.72	23.92
**ED9** 5×IC_{50}	11.48	4.31	27.32	68.38	17.77	41.35	8.89	38.94
**ED12** 1×IC_{50}	11.00	4.79	28.50	66.70	17.19	13.49	12.43	33.92
**ED12** 5×IC_{50}	16.07	22.95	44.26	32.79	14.18	47.08	7.46	8.14

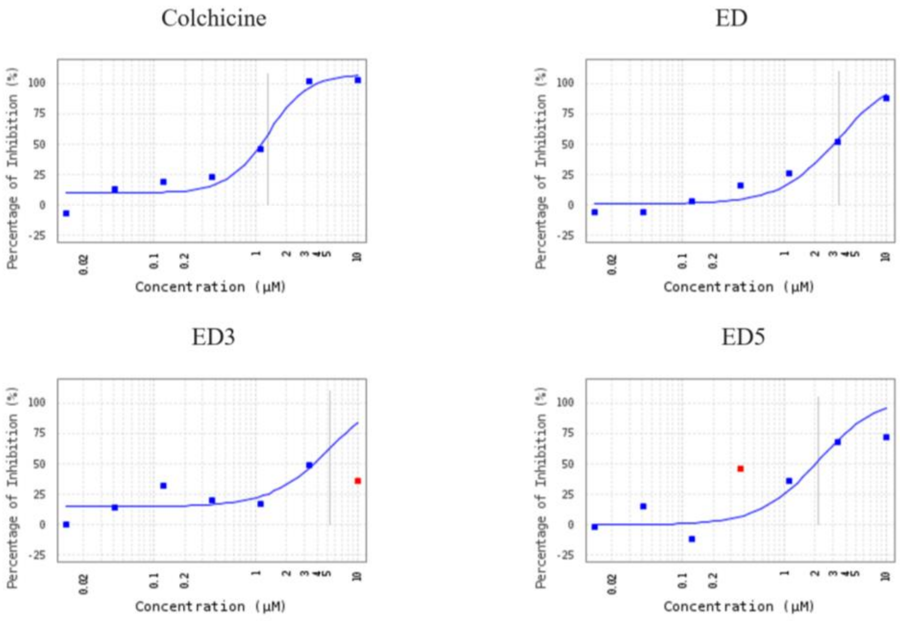

Figure S49. Inhibition of tubulin assembly in dose-response. IC_{50} values and SD were calculated from 2 independent experiments.

3. *In silico* modeling

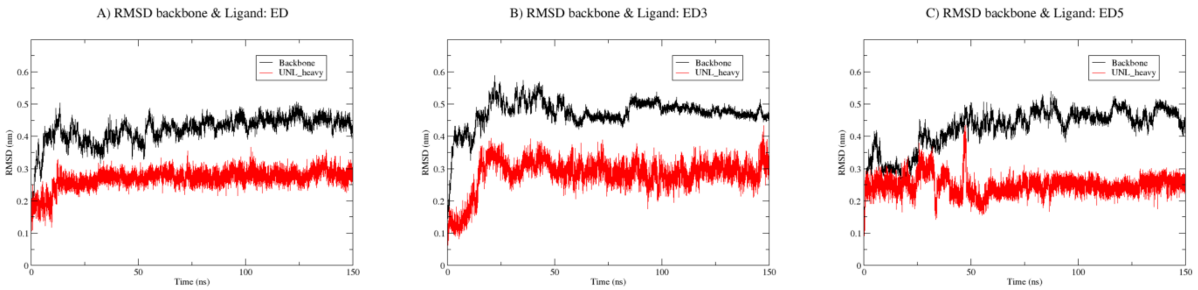

Figure S50. RMSD of tubulin backbone and ED, ED3 and ED5 ligands during 150 ns MD simulations.

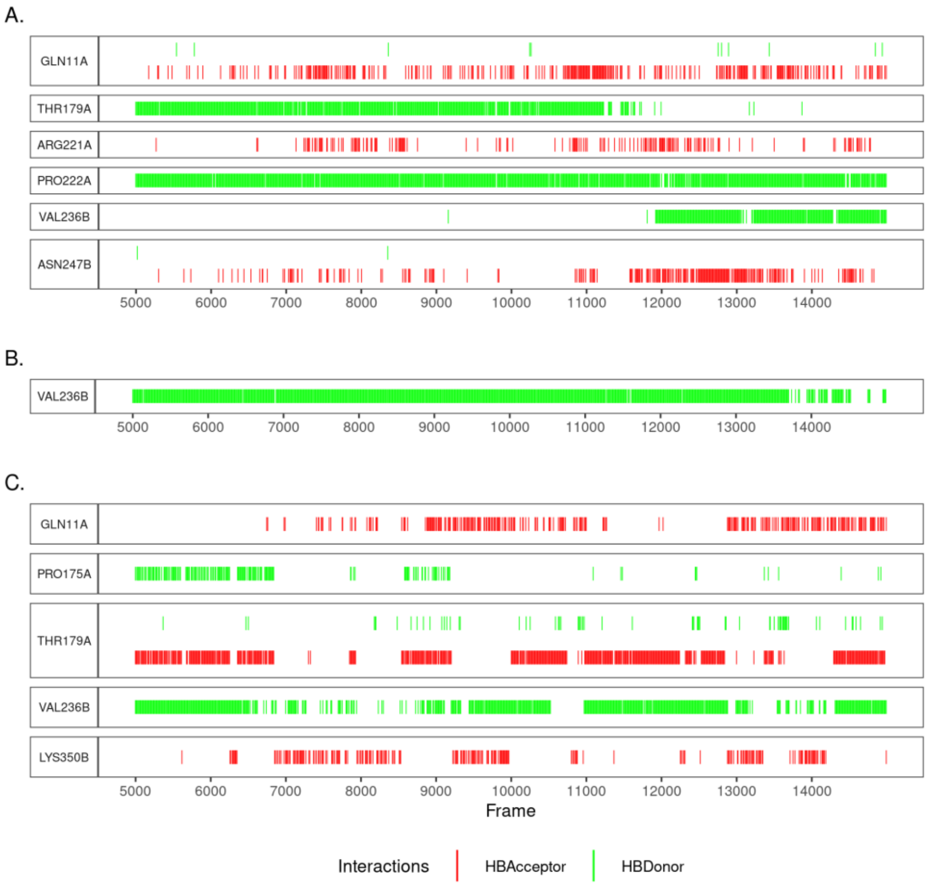

Figure S51. Polar contacts observed during MD simulations of tubulin complexes with ED (A), ED3 (B) and ED5 (C). Since trajectories were stabilized after 50 ns, only polar contacts from 50 to 150 ns having at least 10% occurrence were shown.
